# H_2_S Regulation of Metabolism in Cucumber in Response to Salt-Stress Through Transcriptome and Proteome Analysis

**DOI:** 10.3389/fpls.2020.01283

**Published:** 2020-08-19

**Authors:** Jinglong Jiang, Xuming Ren, Li Li, Ruping Hou, Wang Sun, Chengjin Jiao, Ni Yang, Yanxin Dong

**Affiliations:** ^1^ School of Biological Science and Engineering, Shaanxi University of Technology, Hanzhong, China; ^2^ School of Chemical and Environmental Sciences, Shaanxi University of Technology, Hanzhong, China; ^3^ School of Bioengineering and Biotechnology, Tianshui Normal University, Tianshui, China

**Keywords:** *Cucumis sativus* L., transcriptomic, proteomic, hydrogen sulfide, salt stress

## Abstract

In a previous study, we found that H_2_S alleviates salinity stress in cucumber by maintaining the Na^+^/K^+^ balance and by regulating H_2_S metabolism and the oxidative stress response. However, little is known about the molecular mechanisms behind H_2_S-regulated salt-stress tolerance in cucumber. Here, an integrated transcriptomic and proteomic analysis based on RNA-seq and 2-DE was used to investigate the global mechanism underlying H_2_S-regulated salt-stress tolerance. In total, 11,761 differentially expressed genes (DEGs) and 61 differentially expressed proteins (DEPs) were identified. Analysis of the pathways associated with the DEGs showed that salt stress enriched expression of genes in primary and energy metabolism, such as photosynthesis, carbon metabolism and biosynthesis of amino acids. Application of H_2_S significantly decreased these DEGs but enriched DEGs related to plant-pathogen interaction, sulfur-containing metabolism, cell defense, and signal transduction pathways. Notably, changes related to sulfur-containing metabolism and cell defense were also observed through proteome analysis, such as *Cysteine synthase 1*, *Glutathione S-transferase U25-like*, *Protein disulfide-isomerase*, and *Peroxidase 2*. We present the first global analysis of the mechanism underlying H_2_S regulation of salt-stress tolerance in cucumber through tracking changes in the expression of specific proteins and genes.

## Introduction 

Salinization of soil is gradually becoming an earnest threat to world agriculture, affecting approximately 20% of arable irrigated land and consequently leading to the loss of US$ 27.5 billion per annum (Abdel Latef et al., 2019). Salt stress impedes plant growth and development, crop productivity, and geographic distribution by imposing ionic toxicity and perturbing cellular osmotic potential due to excessive accumulation of Na^+^ (Dai et al., 2018). Soil salinity not only affects plant growth and development but also causes severe osmotic stress, toxicity from sodium and chloride ions, and oxidative damage (Zhang et al., 2019). Therefore, plants may develop a sophisticated mechanism to exclude toxic ions to mitigate salt stress and lots of studies have demonstrated that salt-tolerant genotypes may accumulate less Na^+^ through reducing Na^+^ influx into the root, control of Na^+^ xylem loading, Na^+^ retrieval from the xylem, Na^+^ recirculation in the phloem, Na^+^ efflux from the root, intracellular compartmentation of Na^+^ into the vacuoles, and Na^+^ secretion from the leaf, which is a general rationale for combating salt stress (Zhu, 2003). These responses can be modified by small molecules such as plant growth regulators and signaling molecules (Mostofa et al., 2015a).

Sulfur metabolism is connected directly *via* the Met salvage cycle to ethylene- and polyamine-related responses to abiotic stresses (Sauter et al., 2013). Hydrogen sulfide (H_2_S) is a small gas molecule that resides in all kinds of plant tissues and cells and can serve both as physiological and signaling functions. (Rausch and Wachter, 2005). As a gaseous signal molecule, H_2_S functions in seed germination, growth and development, and stress adaptation in plants (Li et al., 2016). L-cysteine desulfhydrase (L-CD, EC 4.4.1.1) and D-cysteine desulfhydrase (D-CD, EC 4.4.1.15) can catalyze the degradation of L-/D-cysteine to produce H_2_S, amine and pyruvate (Li et al., 2016). The L-CD and D-CD were also identified as being mainly responsible for the degradation of cysteine in order to generate H_2_S (Jin et al., 2011). A direct impact of Cys on ABA formation together with the herein identified co-regulation of genes related to ABA synthesis- and S-metabolism revealed a reciprocal regulatory network with the function of ensuring optimal supply with Cys for ABA synthesis to combat salt stress in *Arabidopsis thaliana* (Cao et al., 2014). H_2_S enhances tolerance to salinity during germination of *Medicago sativa* seeds and in barley seedlings (Wang et al., 2012; Chen J. et al., 2015). H_2_S interacts with the nitric oxide (NO) pathway, alters redox homeostasis, and prevents K^+^ loss in seedlings of *Medicago sativa* (Wang et al., 2012; Lai et al., 2014), regulates the Na^+^/K^+^ balance, mineral homeostasis, and oxidative metabolism in rice (Mostofa et al., 2015b) and maintains lower Na^+^ concentrations in wheat seedlings *via* the regulation of nonselective cation channels (NSCCs) and the SOS1 pathway (Deng et al., 2016). Our previous study has shown that exogenous application of NaHS, an H_2_S donor, could ameliorate salt-induced growth inhibition by maintaining the Na^+^/K^+^ balance and by regulating H_2_S metabolism and the oxidative stress response (Jiang et al., 2019). However, these reports about H_2_S regulation of salt-stress tolerance primarily focused on morphological, physiological, and biochemical aspects, and little is known about the molecular mechanism of H_2_S-regulated salt-stress tolerance at the transcriptomic and proteomic levels in cucumber.

The development and application of “omics” technologies, such as genomics, transcriptomics, proteomics, and metabolomics, have resulted in new widely used experimental methods to overcome limitations in crop quality and yield (Ghatak et al., 2017). Meng et al. (2019) revealed jasmonate ZIM-domain protein 7 (JAZ7)-mediated drought tolerance in *Arabidopsis* using the tandem mass tags (TMT)-based quantitative proteomics and targeted metabolomics. The more advanced proteomics technology, such as quantitative phosphoproteomics are used to elucidate the mechanism that ABAinduced1 identifies AT-Hook-Like10 phosphorylation required for stress growth regulation in *Arabidopsis* (Wong et al., 2019). Combining comparative transcriptomic and proteomic analyses is also an effective way to investigate differential activation of response and metabolism pathways at the global level (Li et al., 2019). Transcriptional profiling based on total RNA sequencing is used to analyze gene expression changes in response to various environmental stresses (Chen Q. Z. et al., 2015) and to map the responding pathways (Wang et al., 2018). Since the expression of some genes is regulated only at the translational level, proteomics is also used to identify the specific proteins that are affected by different physiological conditions (Nogueira et al., 2012). Additionally, finding the correlation between transcript and protein levels helps to find the regular pattern of DEGs and DEPs. Even though two-dimensional gel electrophoresis (2-DGE) was introduced more than four decades ago, nowadays, it is still widely used for whole proteome analysis, comparative analysis of proteome changes, biomarker discovery, cancer research, as well as for the identification of protein isoforms and post-translational modifications (Goez et al., 2018).

Cucumber (*Cucumis sativus* L., 2n = 2x = 14) is a widely cultivated plant in the gourd family and also considered as a model species for the study of phloem characteristics, raffinose family oligosaccharide (RFO) metabolism, and sex determination (Jiang et al., 2019). The cucumber genome is relatively small, with an estimated size of 367 Mb (Arumuganathan and Earle, 1991). Our hydroponically based study aids in understanding how plants respond under NaCl-toxicity and how the use of H_2_S is capable of reverting this toxicity at the transcript and protein expression levels. In the present study, an integrated analysis based on RNA sequencing and 2-DE was used to investigate the global mechanism behind H_2_S-regulated salt-stress tolerance at the transcriptomic and proteomic levels. So far, this study is the first to combine transcriptomic and proteomic analysis of the global mechanism of H_2_S-regulated salt-stress tolerance. This study also contributes to the identification of salt-stress tolerance-related genes, particularly those responsive to H_2_S regulation.

## Materials and Methods

### Plant Culture and Treatments

Seeds of cucumber cultivar “Chunxiaqiuwang” were purchased from the Ningyang County Lu-Ming Seed Co., Ltd. (Shandong province). Seed germination and growth conditions followed those we previously described (Jiang et al., 2019). The seedlings of cucumber were maintained in a controlled growth chamber with a light/dark regime of 14/10 h, relative humidity of 70%, temperature of 25℃ and a photosynthetically active radiation (PAR) of 800 μmol.m^−2^.s^−1^. When the first true leaf emerged, uniform and healthy seedlings were selected and divided into three groups for the following treatments: (i) Hoagland’s nutrient solution (as control, C); (ii) Hoagland’s nutrient solution +200 mM NaCl (as salt treatment, S); (iii) Hoagland’s nutrient solution + 200 mM NaCl +15 µM NaHS (as H_2_S treatment, H_2_S). In our previously studies, 5, 10, 15, and 20 µM NaHS was tested for alleviation of NaCl-induced damage, and morphological and physiological assessments showed that exogenous supplementation with 15 µM NaHS was the most effective in boosting cucumber tolerance to NaCl stress (Jiang et al., 2019). Thus, the control (C), 200 mM NaCl (S), and 15 µM NaHS (H_2_S) treatments were chosen to study the transcriptomic and proteomic responses to H_2_S alleviation of salt-stress in cucumber leaves. NaHS was purchased from Sigma (St. Louis, MO, United States) and used as H_2_S donor. NaHS is commonly used as an H2S donor since it dissociates to water produce HS^−^ and Na^+^, and then, combination of HS^−^ with H^+^ produces H_2_S (Lin et al., 2012). NaHS is responsible for induction of stress tolerance, but not Na_2_S, Na_2_SO_4_, NaHSO_4_, Na_2_SO_3_, NaHSO_3_, or CH_3_COONa (Deng et al., 2016). Three independent biological replicates were conducted per treatment (8~10 plants per treatment) under the same experimental conditions. A 10-ml fresh treatment solution was irrigated every other day at 9:00 AM during the whole culture process, and the remaining treatment solution, which was not fully absorbed by plants, was removed. After 7 days of treatment, the leaves from at least eight seedlings of each treatment sample were harvested as three replicate, immediately frozen in liquid nitrogen and then stored at −80°C until RNA and protein extraction.

### RNA Isolation, cDNA Library Preparation, and Sequencing

Total RNA was isolated from each treatment using the RNAplant Plus Reagent DP437 (Tiangen, Beijing, China) according to the manufacturer’s instructions. The concentration and quality of RNA were determined using an Agilent 2100 Bioanalyzer (Agilent Technologies, Santa Clara, CA, USA). Then, the RNA was sent to the Shanghai Personal Biotechnology Cp. Ltd. for RNA-Seq analysis (Personalbio, Shanghai, China).

A total of 20-mg RNA was equally isolated from the each sample for cDNA library construction. Beads with oligo (dT) were used to enrich poly(A) mRNA, and then, fragmentation buffer was added for interrupting mRNA to short fragment (200~300 nt) which were used as templates to synthesize the first-strand cDNA using random hexamer primer, and the second-strand cDNA was synthesized by adding buffer, dNTPs, RNaseH, and DNA polymerase I, respectively. The double-strand cDNA was purified with QiaQuick PCR Purification Kit (Qiagen, Germany) and washed with EB buffer for end repair and single nucleotide. Finally, the cDNA fragments were connected with sequencing adaptors and gel-electrophoresis was used to select cDNA fragments of 200 bp in size as the templates for amplification with PCR. The library products were ready for sequencing analysis *via* Illumina HiSeq™ 2000 (Illumina, San Diego, CA, USA).

### Bioinformatics Analysis

The RNA-Seq raw sequencing data was filtered using the Cutadapt program (http://cutadapt.readthedocs.io/en/stable/). The filtered clean reads were further mapped to the reference genome (Cucumis_sativus.ASM407v2.dna.toplevel.fa, v 2.39), downloaded from the Ensembl database (http://www.ensembl.org/) using Tophat2 (http://tophat.cbcb.umd.edu/), with up to two mismatches allowed. The transcripts were screened for significant changes in abundance between C, S, and H_2_S samples using DESeq (http://www.bioconductor.org/packages/release/bioc/html/DESeq.html) with a condition of |log_2_^fold-change^|> 1.0 and *p*-value < 0.05. Principal Coordinate Analysis (PCA) was performed on distance matrices, and coordinates were used to draw 2D graphical outputs (Lozupone et al., 2007). All DEGs were searched against the Kyoto Encyclopedia of Genes and Genomes database (KEGG, http://www.genome.jp/kegg/pathway.html) to identify the main metabolic pathways and signal transduction pathways of DEGs using Blastall software (Kanehisa and Goto, 2000).

### Protein Extraction, 2-DE, and Gel Staining

Protein extraction was performed using a modified version of the trichloroacetic acid (TCA)-acetone precipitation method described by Wu et al. (2011) (Wu et al., 2011). Protein concentrations were quantified according to the Bradford method (Bradford, 1976). Isoelectric focusing (IEF) was carried out on 18-cm immobilized pH gradient (IPG) linear gradient strips at pH 4–7. IPG strips in a rehydration tray were loaded with 350 μl of protein sample containing 500-μg protein for 12–16 h at room temperature. The IPG strips were then run on an Ettan IPGphor 3 (GE Healthcare, USA) using the following voltage settings: 50 V for 1 h, 100 V for 2 h, 500 V for 1 h, 1,000 V for 2 h, and 2,000 V for 1 h, followed by a gradient of 5,000 V for 1 h and 10,000 V for 2 h, with a subsequent 10,000 V rapid focusing to reach 65,000 V.h. The temperature was maintained at 20°C, with a maximum current of 50 μA per strip. After running the first dimension, IEF strips were incubated for 15 min in 10-ml equilibration buffer containing 6 M urea, 30% (v/v) glycerol, 2% (w/v) SDS, 1.5-ml Tris-HCl (1.5 M, pH 8.8), 0.002% (w/v) bromophenol blue, and 1% (w/v) DTT and then equilibrated with fresh equilibration buffer containing 2.5% iodoacetamide (IAM) for another 15 min.

The equilibrated strips were placed directly onto 12.5% polyacrylamide-SDS slab gels and sealed with 0.5% agarose solution containing bromophenol blue dye. The second dimensional SDS–polyacrylamide gel electrophoresis (SDS-PAGE) was conducted using the Ettan DALTSix electrophoresis system (GE Healthcare, USA). Electrophoresis was carried out at 15 mA per gel for 30 min, and then, 38 mA per gel until the bromophenol blue dye front was approximately 1 cm from the bottom of the gel. After 2-DE, protein spots were visualized using Coomassie Brilliant Blue (CBB) R-250.

### Image and Data Analysis

The 2-DE gels were scanned at 300 dpi using an Image Scanner III (GE Healthcare). Spot detection, quantification, and matching were conducted using ImageMaster™ 2D Platinum version 7.0 analysis software (GE Healthcare). The optimized parameters were: smooth 2.0, min area 5.0, and saliency 3.0. The abundance of each spot was estimated by the percentage volume (%VOL), which was normalized as a percentage of the total volume of all the spots present in the gel to correct for variability caused by loading, gel staining, and destaining. Only spots with significant and biologically reproducible changes (abundance variation ≥1.3-fold or ≤0.67-fold, one way ANOVA, *p* ≤ 0.05) were considered to be differentially expressed proteins (DEPs).

### In-Gel Digestion, MALDI-TOF/TOF-MS, and Database Searching

The selected DEPs were manually excised from the 2-DE gels, and gel pieces were digested overnight in trypsin at 37°C. The hydrolysates were then transferred to new tubes and disrupted by sonication for 15 min in a buffer containing 100-μl 60% acetonitrile (ACN)/0.1% trifluoroacetic acid (TFA) and desalinated in a ziptip (Millipore). Peptides in the supernatant were collected by centrifugation. The peptide pellets were dissolved with 1-μl matrix solution. After drying completely at room temperature, 0.6-μl over-saturated CHCA (50% ACN/0.1% TFA) was added. MS and MS/MS data for protein identification were obtained by using a MALDI-TOF-TOF instrument with an AB SCIEX MALDI TOF-TOF™ 5800 Analyzer (AB SCIEX, Foster City, CA, USA) equipped with a neodymium (Nd): yttrium-aluminum-garnet (YAG) laser (laser wavelength: 355 nm). Instrument parameters were set using the 4000 Series Explorer software (Applied Biosystems). The MS spectra were recorded in reflector mode in a mass range from 800 to 4000 with a focus mass of 2000. The TOF/TOF calibration mixtures (AB SCIEX) were used to calibrate the spectrum to a mass tolerance within 10 ppm. The MS spectra were processed using TOF-TOF Series Explorer software (V4.0, AB SCIEX). At least 1,000 laser shots were typically accumulated with a laser pulse rate of 400 Hz in the MS mode, whereas in the MS/MS mode spectra, up to 2,000 laser shots were acquired and averaged with a pulse rate of 1,000 Hz. For MS calibration, autolysis peaks of trypsin ([M^+^H]^+^ 842.5100 and 2, 211.1046) were used as internal calibrates, and the most intense ion signals (up to 10) were selected as precursors for MS/MS acquisition, excluding the trypsin autolysis peaks and the matrix ion signals. Peptide mass finger printing (PMF) and MS/MS queries were performed by using the MASCOT search engine 2.2 (Matrix Science, Ltd. UK) embedded into GPS-Explorer Software 3.6 (Applied Biosystems) on the database of uniprot_Eimeria acervulina with the following parameter settings: 100 ppm mass accuracy, trypsin cleavage one missed cleavage allowed, carbamidomethylation set as fixed modification, oxidation of methionine was allowed as variable modification, MS/MS fragment tolerance was set to 0.4 Da. All proteins with a confidence interval (CI) > 95% were considered to be identified successfully.

### Bioinformatics Analysis

The DEPs were classified according to the biological processes in which they are involved based on UniProtKB (http://www.uniprot.org/) and NCBI (https://www.ncbi.nlm.nih.gov/) databases. Hierarchical clustering of protein expression patterns was performed using MultiExperiment Viewer (MeV) software version 4.9. The relative ratios of DEPs were subjected to log^2^ transformation, the Euclidean distance similarity metric was used to define the similarity, and hierarchical clusters were assembled using the complete linkage clustering method. The clustering result was visualized through MeV software.

### Statistical Analyses

Data for RNA data and protein expression levels was analyzed using SPSS software (version 21.0, SPSS Inc., USA). Differences in proteins expression levels were analyzed by Duncan’s multiple range test at *P* ≤ 0.05 level of significance.

### Data Deposition

Raw transcriptome data from this manuscript can be found in the NCBI BioProject database under accession numbers: SRP220380.

## Results

### Screening Significant Differential Transcripts From Transcriptomes Based on RNA-Seq

Total RNA from three replicates each of the control (C), 200 mM NaCl (S), and 15 µM NaHS (H_2_S) treatment samples was extracted for RNA sequencing by Illumina Hiseq 2500. Nine libraries was constructed and searched for DEGs associated with H_2_S-regulated salt-stress tolerance. After filtering raw reads, a total of 382,202, 188 high-quality reads were obtained from the nine samples. The clean reads of the sequencing results for each cDNA library accounted for >97.42% of the raw reads (Q_30_ > 86.22%). The standard deviation of clean reads among the three replicates for each treatment was ~0.2% (**Table S1**). Among all nine samples, 85.75~90.78% of the reads could be mapped to the genome of cucumber. Of these mapped reads, 84.79~89.90% matched uniquely.

### H_2_S Treatment Changed DEGs Pattern in Cucumber Under Salt Stress

**Figure 1:**
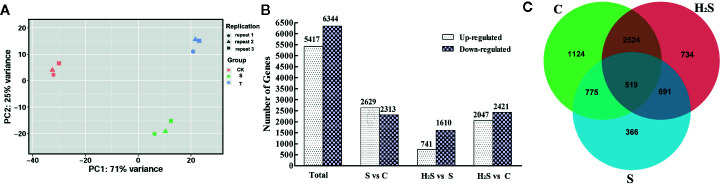
Identification and analysis of DEGs in the cucumber leaf samples of C, S, and H_2_S. **(A)** The similarity of the expression of genes was compared in three sample groups by using PCA. The top 500 genes with the highest contribution were chosen for PCA analysis. **(B)** Comparison analysis of DEGs in the S vs. C, H_2_S vs. S, and H_2_S vs. C were shown in column chart. **(C)** Common or unique DEGs were compared using the Venn diagram.

The similarity between the expression of genes between the cucumber leaf samples following C, S, and H_2_S treatment was determined by PCA. As shown in **Figure 1**, the genes identified in the C, S, and H_2_S samples were clearly separated from each other, and the difference was statistically significant. The expressed genes in the control and H_2_S samples clustered more closely to each other than those from the salt treatment, indicating that salt stress remarkably changes gene expression and that H_2_S may alleviate some of these changes at the transcript level. The principal coordinates PC1 and PC2 represented 71% and 25% of the variance, respectively, and the contribution of the cumulative variance of the two principal coordinates (PC1 and PC2) accounted for 96% (**Figure 1**). The transcripts were compared between C and S, between H_2_S and S, and between C and H_2_S by DESeq. Transcripts with a *P* < 0.05, and |log_2_^fold change^| > 1 were defined as DEGs. All identified DEGs are shown in **Table S2**. A total of 11,761 (5,417 up-regulated and 6,344 down-regulated) DEGs were detected from the C, S, and H_2_S samples (**Figure 1**). Among these DEGs, 4,942 DEGs (2,629 up-regulated and 2,313 down-regulated) in S vs. C, 2,351 DEGs (741 up-regulated and 1,610 down-regulated) in H_2_S vs. S, and 4,468 DEGs (2, 047 up-regulated and 2, 421 down-regulated) in H_2_S vs. C were detected (**Figure 1**). A Venn diagram of the genes show how many genes are specific to one treatment and how many genes transcriptionally respond to multiple treatments (**Figure 1**). Of the 11,761 DEGs, 1,124 DEGs were unique to untreated plants, 366 genes were unique to salt treatment, and 734 were unique to H_2_S and S treatment (**Figure 1**). Additionally, the C, S, and H_2_S treatment samples shared 519 DEGs as shown in **Figure 1**.

### H_2_S Treatment Changed GO (Gene Ontology) Enrichment in Cucumber Under Salt Stress

**Figure 2:**
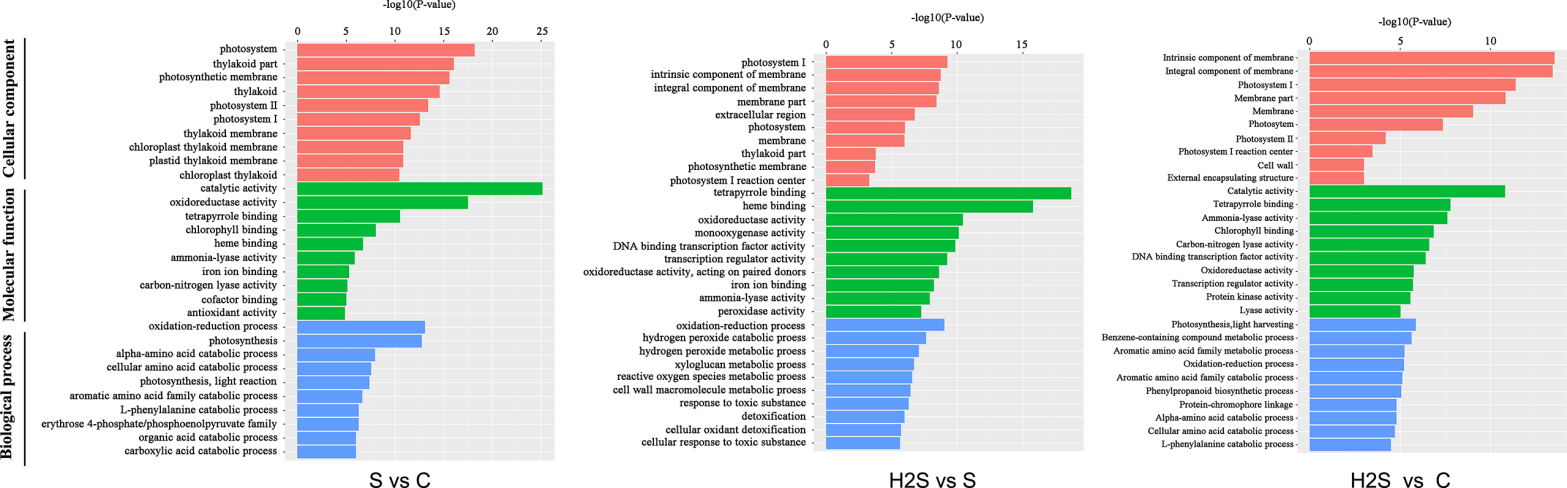
Histogram of gene ontology (GO) classification. The results are summarized in three main categories: biological process, cellular component, and molecular function.

After gene annotation, GO enrichment analysis of differentially expressed genes was carried out. As shown in **Figure 2**, photosystem took the first place in cellular components in the S vs. C and H_2_S vs. S, and intrinsic component of membrane took the first place in cellular components in the H_2_S vs. C. Catalytic activity was the largest category in molecular function in the S vs. C and H_2_S vs. C; however, tetrapyrrole binding was the largest category in molecular function in the H_2_S vs. S (**Figure 2**). Oxidation-reduction process occupied the largest proportion of biological processes in the S vs. C and H_2_S vs. S, and photosynthesis, light harvesting occupied the largest proportion of biological processes in the H_2_S vs. C (**Figure 2**).

### H_2_S Treatment Changed KEGG Enrich Pattern of DEGs in Cucumber Under Salt Stress

**Figure 3:**
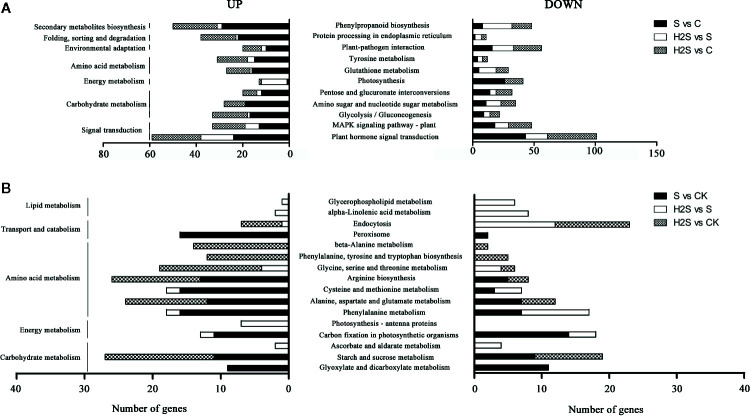
KEGG pathway enrichment analysis of DEGs. The top 20 pathways with highest enrichment level were exhibited according to the amount and enrichment level of DEGs annotated in the S vs. C, H_2_S vs. S, and H_2_S vs. C respectively. **(A)** The common pathways in the S vs. C, H_2_S vs. S, and H_2_S vs. C are exhibited. **(B)** The special pathways in the S vs. C or H_2_S vs. S or H_2_S vs. C are exhibited.

The DEGs were compared to known metabolic and signaling pathways to identify those that were more responsive to salt stress and H_2_S amelioration. In the transcriptome, there were 118 KEGG pathways differentially regulated in the S vs. C samples, 111 in the H_2_S vs. C, and 88 in the H_2_S vs. S (**Table S3**). The 20 most enriched pathways, based on the number of DEGs (including up- and down-regulated genes), are shown in **Figure 2**. The signal transduction, carbohydrate metabolism, energy metabolism, amino acid metabolism, environmental adaptation, folding, sorting and degradation, and secondary metabolites biosynthesis pathways are involved in the three pairwise comparisons (S vs. C, H_2_S vs. C, and H_2_S vs. S), and the detailed metabolic pathway are as follows: plant hormone signal transduction, MAPK signaling pathway, glycolysis/gluconeogenesis, amino sugar and nucleotide sugar metabolism, pentose and glucuronate interconversions, photosynthesis, tyrosine metabolism, glutathione metabolism, plant-pathogen interaction protein processing in endoplasmic reticulum, and phenylpropanoid biosynthesis (**Figure 3**). Salt stress significantly resulted in the changes of basal metabolism such as carbohydrate metabolism (starch and sucrose metabolism and glyoxylate and dicarboxylate metabolism), energy metabolism (carbon fixation in photosynthetic organisms), amino acid metabolism (arginine biosynthesis, cysteine and methionine metabolism, alanine, aspartate and glutamate metabolism and phenylalanine metabolism), and transport and catabolism (peroxisome) in S vs. C (**Figure 3**). However, the treatment of H_2_S regulated the lipid metabolism (alpha-Linolenic acid metabolism and glycerophospholipid metabolism), and energy metabolism (photosynthesis-antenna proteins), as well as amino acid metabolism in leaves of cucumber under salt stress (H_2_S vs. S) (**Figure 3**). In addition, the treatment of H_2_S alone regulated the genes involved in the amino acid metabolism, carbohydrate metabolism, and transport and catabolism (H_2_S vs. C) (**Figure 3**).

### H_2_S Treatment Changed Protein Expression Profiling in Cucumber Under Salt Stress

Proteomics analysis using 2-DE for protein separation was performed to examine changes in protein levels in cucumber leaves and representative gel maps are shown in **Figure S1**. The total numbers of protein spots reproducibly detected using ImageMaster™ 2D Platinum software were 875 ± 93, 986 ± 11, and 776 ± 47 in the S vs. C, H_2_S vs. S, and H_2_S vs. C, respectively. Quantitative image analyses revealed a total of 69 protein spots showing significant changes in their abundance in pairwise comparisons (*P* < 0.05). Among the 69 DEPs observed in the gels, there were 29 DEPs in S vs. C, 25 DEPs in H_2_S vs. S, and 15 DEPs in H_2_S vs. C. Finally, 61 DEPs were chosen for further identification using MALDI-TOF/TOF MS (**Figure S1A**) after removing eight very low abundance proteins. Of these 61 DEPs, 42 proteins were up-regulated and 19 proteins were down-regulated, in pairwise comparisons (**Figure S1B**).

### MALDI-TOF/TOF-MS Analysis and Identification of DEPs

Altogether, 49 DEPs were successfully identified by MALDI-TOF/TOF-MS. To investigate the functional information of these proteins, the Viridiplantae (taxonomy, Green plant) data in NCBInr and Uniprot Swissprot were searched using the Mascot search engine. The basic identification information for each of these DEPs was listed in **Table S4** and summarized in **Table 1**. It was interesting to note that a chloroplast stem-loop binding protein of 41 kDa (spots 14 and 17) and an acetylornithine deacetylase-like protein (spots 31, 32, and 51) were identified in more than one spot on the same gel. This multiple observation of the same protein could result from the presence of different protein isoforms, post-translational modification, or degradation. Forty-seven proteins out of the 49 identified MS-sequenced proteins (except spots 1 and 4) were matched with proteins in *Cucumis sativus* L.

**Table 1:** Cucumber leaf proteins responsive to salt and/or H2S treatment and identified by MALDI-TOF/TOF-MS.

Spot no.^a^	Accession no.^b^	Protein name	Score	CI (%)^c^	Peptidesmatched	TMr (kDa)/TpI^d^	EMr (kDa)/EpI^e^	SC(%)^f^	Species	T test *p*-value	Change folds in intensity^g^
Photosynthesis											
1(↑)	O65194	Ribulose bisphosphate carboxylase small chain, chloroplastic	65	82.41	11	20.238/8.86	96.846/4.47	50	Medicago sativa	0.049	6.41
6(↓)	XP_004144412.1	Phosphoribulo kinase, chloroplastic	574	100	22	46.497/5.97	41.667/5.40	66	C. sativus	0.009	0.52 ± 0.05
11(↑)	XP_004141091.2	Carbonic anhydrase 2 isoform X2	75	99.85	2	35.702/6.19	30.455/6.02	7	C. sativus	0.009	2.21 ± 0.05
12(↓)	XP_004137418.1	Ribulose-phosphate 3-epimerase, chloroplastic	282	100	8	30.165/8.62	26.364/6.07	38	C. sativus	0.022	0.42 ± 0.06
13(↓)	ABN41481.1	Putative chloroplast ribulose-1,5-bisphosphate carboxylase/oxygenase small subunit, partial	267	100	7	10.907/5.24	16.582/6.31	54	C. sativus	0.035	0.23 ± 0.14
14(↓)	XP_004140080.1	Chloroplast stem-loop binding protein of 41 kDa a, chloroplastic	437	100	18	44.502/6.57	36.000/6.50	49	C. sativus	0.015	0.62 ± 0.01
17(↑)	XP_004140080.1	Chloroplast stem-loop binding protein of 41 kDa a, chloroplastic	57	90.58	10	44.502/6.57	35.667/6.70	24	C. sativus	0.017	12.34
30(↑)	XP_004142574.1	Rubisco accumulation factor 1, chloroplastic	116	100	14	49.424/4.98	44.500/4.79	29	C. sativus	0.016	2.14 ± 0.20
37(↑)	XP_011650288.1	Ferredoxin–NADP reductase, leaf isozyme, chloroplastic isoform X2	735	100	20	40.299/8.54	32.364/6.56	57	C. sativus	0.041	1.96 ± 0.41
40(↑)	XP_004138462.1	Ribulose bisphosphate carboxylase, chloroplastic	425	100	8	51.768/5.58	44.487/4.82	26	C. sativus	0.042	2.29 ± 0.60
43(↑)	NP_001267658.1	Sedoheptulose-1,7-bisphosphatase, chloroplastic-like	673	100	26	42.075/5.96	37.564/4.93	62	C. sativus	0.048	1.79 ± 0.25
52(↑)	XP_004148579.1	Chlorophyll a-b binding protein of LHCII type 1-like	286	100	6	28.299/5.47	33.727/5.22	28	C. sativus	0.005	2.81 ± 0.02
Protein folding, assembly and degradation											
3(↑)	XP_004149798.1	Protein disulfide-isomerase	670	100	35	57.046/4.88	65.084/4.89	68	C. sativus	0.046	1.91 ± 0.17
4(↑)	Q9SAD7	Eukaryotic translation initiation factor 4B2	65	81.58	19	59.289/8.69	34.091/4.64	32	Arabidopsis thaliana	0.024	3.63 ± 0.89
5(↑)	XP_004139931.1	26S protease regulatory subunit 6B homolog	409	100	19	46.911/5.42	55.712/5.57	47	C. sativus	0.033	1.75 ± 0.20
9(↑)	XP_004154254.1	Probable carboxylesterase 13	132	100	8	35.086/5.70	37.500/5.79	28	C. sativus	0.014	5.47 ± 2.32
23(↑)	XP_004141139.1	T-complex protein 1 subunit theta	92	99.996	16	58.815/5.44	62.130/5.57	35	C. sativus	0.040	1.95 ± 0.28
39(↓)	XP_004141034.2	Mitochondrial-processing peptidase subunit alpha	77	99.892	8	54.059/5.62	54.328/5.50	16	C. sativus	0.031	0.63 ± 0.08
42(↑)	XP_004146811.1	Eukaryotic initiation factor 4A-8	394	100	17	46.830/5.37	42.436/5.38	42	C. sativus	0.041	1.95 ± 0.22
48(↑)	XP_011654558.1	Peptidyl-prolyl cis-trans isomerase A1	339	100	11	27.374/8.11	18.950/5.45	46	C. sativus	0.010	1.20 ± 0.03
55(↑)	XP_004141710.1	Proteasome subunit alpha type-6	109	100	13	27.279/5.74	27.182/6.02	53	C. sativus	0.006	1.81 ± 0.02
61(↑)	XP_004141688.1	Eukaryotic initiation factor 4A-11	138	100	8	46.797/5.38	44.590/5.37	22	C. sativus	0.011	1.11 ± 0.00
Amino acid biosynthesis											
20(↑)	XP_011652060.1	Elongation factor 1-delta isoform X1	274	100	9	24.670/4.45	31.087/4.48	46	C. sativus	0.019	1.64 ± 0.05
28(↓)	XP_004136850.1	Ketol-acid reductoisomerase, chloroplastic	348	100	15	63.533/6.22	63.486/5.82	30	C. sativus	0.031	0.64 ± 0.08
31(↓)	XP_004148484.1	Acetylornithine deacetylase-like	633	100	15	48.515/4.83	43.333/4.91	42	C. sativus	0.004	0.63 ± 0.02
32(↓)	XP_004148484.1	Acetylornithine deacetylase-like	748	100	18	48.515/4.8.3	42.333/4.86	50	C. sativus	0.050	0.44 ± 0.10
51(↑)	XP_004148484.1	Acetylornithine deacetylase-like	895	100	21	48.515/4.83	43.333/4.94	57	C. sativus	0.031	6.64
56(↑)	XP_004138495.1	Cysteine synthase 1, mitochondrial	142	100	14	40.323/7.71	34.091/6.55	40	C. sativus	0.005	1.56 ± 0.00
Carbohydrate and energy metabolism											
7(↑)	XP_004145948.1	Probable 6-phosphogluconolactonase 4, chloroplastic	83	99.97	6	34.655/6.11	28.636/5.37	27	C. sativus	0.044	5.71
8(↑)	XP_004136700.2	Secoisolariciresinol dehydrogenase-like	202	100	8	29.089/5.72	29.545/5.63	32	C. sativus	0.015	3.78 ± 0.04
10(↑)	NP_001267500.1	Alpha-galactosidase-like precursor	131	100	9	45.668/5.60	49.686/5.96	34	C. sativus	0.007	2.33 ± 0.04
24(↑)	XP_004147146.1	Malate dehydrogenase, cytoplasmic	374	100	13	35.745/5.76	38.333/5.68	47	C. sativus	0.043	1.51 ± 0.18
25(↑)	YP_004849346.1	ATPase subunit 1, mitochondrion	901	100	22	54.959/5.58	53.650/5.74	50	C. sativus	0.009	1.34 ± 0.05
26(↑)	XP_004143301.1	Enolase isoform X1	810	100	17	47.714/5.48	58.568/5.59	51	C. sativus	0.049	1.93 ± 0.34
27(↑)	XP_004149141.2	Inositol-3-phosphate synthase-like	525	100	22	56.342/5.49	60.264/5.77	45	C. sativus	0.014	1.16 ± 0.03
45(↑)	XP_011656794.1	Pyruvate dehydrogenase E1 component subunit beta-1, mitochondrial	455	100	11	39.627/5.60	33.551/5.28	40	C. sativus	0.033	1.47 ± 0.14
47(↓)	XP_004152516.1	Glyceraldehyde-3-phosphate dehydrogenase, cytosolic-like	878	100	19	36.604/6.25	34.275/6.56	76	C. sativus	0.007	0.75 ± 0.01
49(↑)	Q4VZG9	ATP synthase epsilon chain, chloroplastic	547	100	9	14.766/5.39	15.067/5.20	71	C. sativus	0.041	1.18 ± 0.01
60(↑)	BAJ23911.1	Cytosolic alkenal/one oxidoreductase	192	100	9	34.401/5.48	38.333/5.47	43	C. sativus	0.045	1.50 ± 0.12
Secondary metabolite biosynthesis											
21(↑)	XP_004143844.1	Phosphoribosylformylglycinamidine cyclo-ligase, chloroplastic/mito- chondrial	123	100	11	42.779/5.50	32.536/4.85	35	C. sativus	0.046	1.48 ± 0.11
57(↓)	XP_004137828.1	29 kDa ribonucleoprotein, chloroplastic	60	94.326	4	30.480/5.84	24.175/4.70	16	C. sativus	0.048	0.62 ± 0.06
Stress and defense											
33(↑)	XP_004147353.1	Actin-7	281	100	11	41.683/5.31	42.500/5.51	41	C. sativus	0.039	1.77 ± 0.23
36(↓)	XP_004142149.1	Peroxidase 2	261	100	8	34.822/6.43	33.636/5.91	26	C. sativus	0.031	0.14 ± 0.14
41(↓)	AAA33129.1	Peroxidase	695	100	11	34.276/4.94	36.795/4.50	43	C. sativus	0.046	0.37 ± 0.10
46(↓)	XP_004147762.1	Glutathione S-transferase U25-like	352	100	13	25.057/5.18	24.010/5.10	38	C. sativus	0.035	0.71 ± 0.02
Other and unknown proteins											
29(↓)	KGN63436.1	Hypothetical protein Csa_1G000600	82	99.97	21	95.949/5.95	76.926/5.65	24	C. sativus	0.012	0.66 ± 0.02
38(↓)	XP_004136034.1	Uncharacterized protein OsI_027940	65	98.44	7	22.557/4.45	24.780/4.44	37	C. sativus	0.043	0.59 ± 0.10
44(↓)	KGN54020.1	Hypothetical protein Csa_4G268040	776	100	18	37.807/6.22	30.797/5.13	53	C. sativus	0.006	0.90 ± 0.01
58(↑)	KGN54259.1	Hypothetical protein Csa_4G296130	634	100	12	35.144/8.98	27.029/5.75	41	C. sativus	0.014	1.24 ± 0.02

↑ and ↓: Up-regulated expression and Down-regulated expression, respectively.

^a^Numbering corresponds to the 2-DE in **Figure S1**.

^b^Accession number from the NCBInr and SwissProt database.

^c^Protein score Confidence Interval.

^d^TMr and TpI are theoretical molecular mass and theoretical isoelectric point, respectively.

^e^EMr and EpI are experimental molecular mass and experimental isoelectric point, respectively.

^f^The sequence coverage of identified proteins.

^g^Data represents the mean of two independent replicates with standard error as indicated in **Table S4**.

### H_2_S Treatment Changed Functional Categorization of the DEPs in Cucumber Under Salt Stress

Salt stress resulted in 22 DEPs, 17 of which were up-regulated and 5 of which were down-regulated in the S vs. C sample (**Figure S2A**). However, H_2_S treatment of cucumber induced only 8 DEPs, 7 of which were up-regulated and only one of which was down-regulated in the H_2_S vs. C sample, and 19 proteins were significantly altered (*P* < 0.05) in the H_2_S vs. S sample, with 9 proteins showing up-regulation and 10 proteins showing down-regulation (**Figure S2A**). Functional categorization of the DEPs in S vs. C showed that 31.82% of the DEPs participated in photosynthesis and carbohydrate and energy metabolism, followed by protein folding, assembly and degradation (22.73%), amino acid biosynthesis (9.09%), and secondary metabolite biosynthesis (4.08%) (**Figure S2B**). In the H_2_S vs. S, 21.05% of the DEPs belonged to stress and defense-related protein and another 21.05% to photosynthesis, followed by protein folding, assembly and degradation (15.79%), carbohydrate and energy metabolism (15.79%), and amino acid biosynthesis (10.53%). In the H_2_S vs. C, 25% of the DEPs participated in amino acid biosynthesis and protein folding, assembly and degradation, followed by photosynthesis, secondary metabolite biosynthesis, and carbohydrate and energy metabolism (**Figure S2B**). Based on their putative physiological and biochemical functions, the majority of these DEPs belonged to one of seven categories, namely, photosynthesis (12, 24.49%), carbohydrate and energy metabolism (11, 22.45%), protein folding, assembly and degradation (10, 20.41%), amino acid biosynthesis (6, 12.24%), secondary metabolite biosynthesis (2, 4.08%), stress and defense (4, 8.16%), and other and unknown proteins (4, 8.16%) in all treatment samples (**Figure S2B**).

### H_2_S Treatment Changed KEGG Enrich Pattern of DEPs in Cucumber Under Salt Stress

There were 49 KEGG metabolic pathways that were enriched for DEPs responding to S and H_2_S treatments (**Table S5-a**), and most of DEPs were enriched in 13 pathways (**Table S5-b**, **Figure S3**). The primary metabolism-related pathways, such as photosynthesis (ko00710, 7), energy metabolism (ko00010, 6), and amino acid biosynthesis (ko00940, 4) were enriched in H_2_S vs. S. It is noteworthy that several DEPs were enriched in the sulfur and cysteine metabolism pathways (ko00270, 3). These pathways are responsible for the synthesis of endogenous H_2_S, suggesting that exogenous H_2_S treatment might cause changes in endogenous H_2_S production along with altered regulation of other signaling pathways. However, very few other DEPs were enriched in other primary metabolism-related pathways, only nitrogen metabolism (ko00910, 1 DEP), lipid metabolism (ko00603, 1), and purine metabolism (ko00230, 1). Additionally, some DEPs were enriched in defense-related pathways, such as glutathione metabolism (ko00480, 1), inositol phosphate metabolism (ko00562, 1), protein processing in ER (endoplasmic reticulum) (ko04141, 1), proteasome (ko03050, 2), and RNA transport (ko03013, 1).

### The Correlation Analysis of Differently Expressed Genes and Proteins

**Figure 4:**
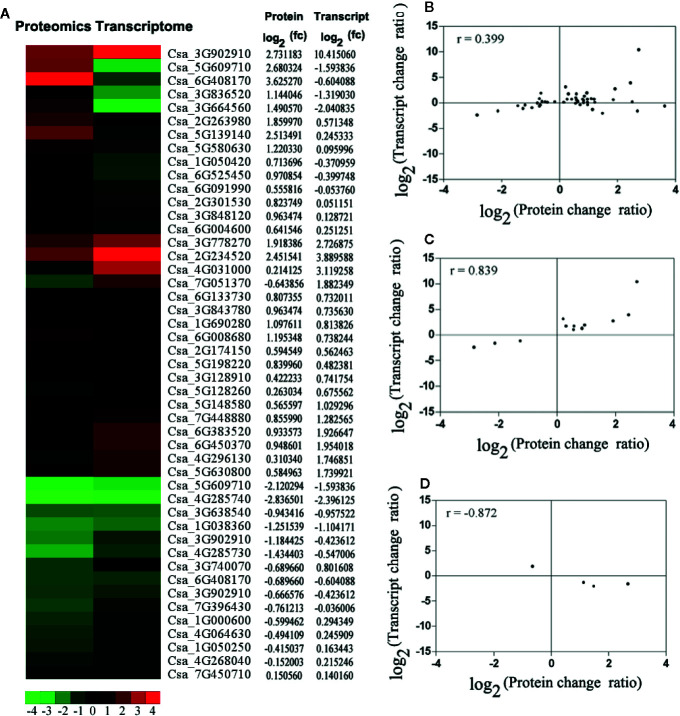
The correlation analysis based on proteomics and transcriptome. **(A)** The expression pattern clustering analysis for 47 differential expression mRNAs and proteins with correlations. fc: fold of change. Red represents up-regulated and green represents down-regulated. **(B)** The correlation analysis of all quantitative protein and gene expression level. **(C)** The correlation analysis of differential expression proteins and genes, which had the same changing trend. **(D)** The correlation analysis of significant differential expression both proteins and genes, which had the opposite changing trend. For the correlation analysis diagram **(B–D)**, the level of differential expression proteins was labeled on the horizontal axis, and the level of gene expression was labeled on the vertical.

To correlate transcript and protein expression profiles, accession numbers from the proteome were extracted and compared with the annotated RNA-Seq libraries (**Table S6**). The expression levels of 17 out of the 47 proteins were consistent with the mRNA levels, indicating that only a few proteins were regulated directly at the transcriptional level. Cluster analyses of the 47 differentially expressed genes and proteins were conducted (**Figures 4A**). Then, comparative analyses of 12 genes (27.66%) that showed similar regulation at both the transcript and protein levels and 4 genes (8.51%) that showed opposite at the transcript and protein levels was conducted (**Figures 4C**). The Pearson Correlations (r) were calculated to be 0.399 (**Figure 4**), 0.839 (**Figure 4**), and −0.872 (**Figure 3**).

### Integrated Pathway Analysis During H_2_S Regulation of Salt-Stress Tolerance in Cucumber

**Figure 5:**
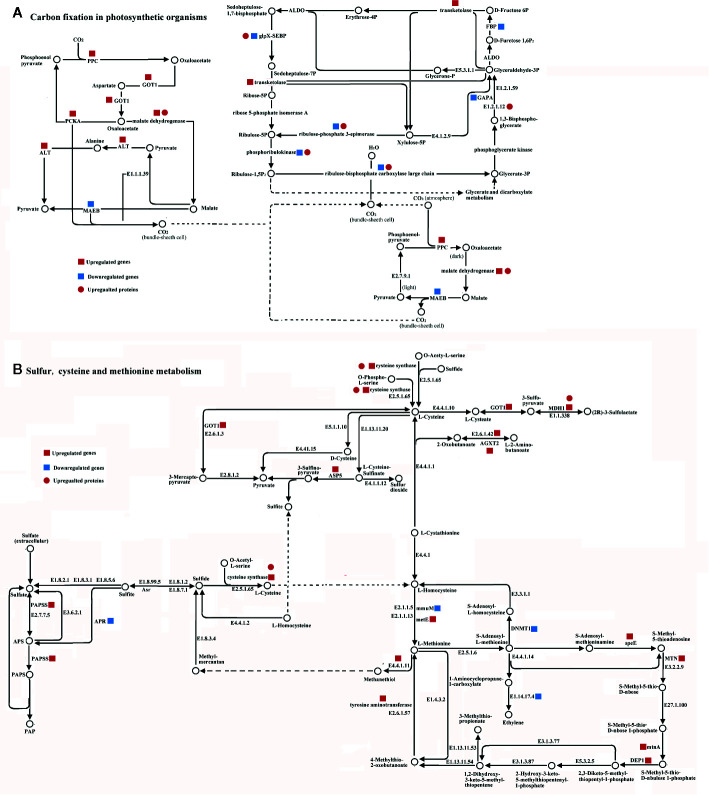
Integrated pathway of carbon fixation in photosynthetic metabolism pathway **(A)** and sulfur, cysteine, and methionine metabolism pathway **(B)** involved in the H_2_S regulation salt-stress tolerance based on DEGs and DEPs identified in leaves of cucumber. Genes and proteins were mapped on pathway of carbon fixation in photosynthetic or sulfur, cysteine and methionine metabolism using KEGG database, respectively. Red square, blue square, and red circle indicate up-regulated genes, down-regulated genes, and up-regulated proteins, respectively. Abbreviations are as follows: AGXT2, alanine-glyoxylate transaminase; ALT, alanine transaminase; APR, adenylyl-sulfate reductase; ASP5, aspartate aminotransferase; DEP1, methylthioribulose 1-phosphate dehydratase; DNMT1, DNA (cytosine-5)-methyltransferase1; FBP, fructose-1,6-bisphosphatase I; GAPA, glyceraldehyde-3-phosphate dehydrogenase (NADP+); GOT1, glutamic oxaloacetic transaminase; MAEB, malate dehydrogenase (oxaloacetate-decarboxylating)(NADP+); MDH1, malate dehydrogenase; metE, 5-methyltetrahydropteroyltriglutamate-homocysteine methyltransferase; MTN, 5’-methylthioadenosine nucleosidase; mtnA, methylthioribose-1-phosphate isomerase; PPC, Phosphoenolpyruvate carboxylase; PCKA, phosphoenolpyruvate carboxykinase(ATP); speE, spermidine synthase.

Forty DEG/DEPs could be mapped to just two metabolic pathways within cucumber (**Figure 5**). Among them, 13 DEGs (6 up-regulated genes and 7 down-regulated genes) and 7 DEPs (7 up-regulated proteins) were mapped onto carbon fixation in photosynthetic metabolism using the KEGG database (**Figure 5**). Nine of the transcripts, *Phosphoenolpyruvate carboxylase* (PPC), *malate dehydrogenase*, *glutamic oxaloacetic transaminase* (GOT1), *alanine transaminase* (ALT), *transketolase*, *malate dehydrogenase (oxaloacetate-decarboxylating)(NADP^+^)* (MAEB), *phosphoenolpyruvate carboxykinase* (ATP) (PCKA), *glyceraldehyde-3-phosphate dehydrogenase (NADP^+^)* (GAPA), and *fructose-1*,*6-bisphosphatase I* (FBP), are mainly related to carbon fixation (**Figure 5A**). Meanwhile, malate dehydrogenase, ribulose-bisphosphate carboxylase large chain, ribulose-phosphate 3-epimerase, phosphoglycerate kinase, and sedoheptulose-1,7-bisphosphatase showed significant up-regulated at the proteome level. Among these, malate dehydrogenase showed the same up-regulated pattern at both the transcriptome and proteome levels (**Figure 5**). However, ribulose-bisphosphate carboxylase large chain, ribulose-phosphate 3-epimerase, phosphoglycerate kinase, and sedoheptulose-1,7-bisphosphatase showed a down-regulation in the transcriptome and an up-regulation in the proteome (**Figure 5**).

Twenty of the DEGs could be mapped to the sulfur, cysteine and methionine metabolism pathways, all of which play important roles in H_2_S regulation (**Figure 5**). Eighteen DEGs (14 up-regulated genes and 4 down-regulated genes) and 2 DEPs (2 up-regulated proteins) were mapped onto sulfur, cysteine, and methionine metabolism using the KEGG database. *Cysteine synthase*, *S-adenosylmethionine decarboxylase*, *spermidine synthase* (speE), *methylthioribulose 1-phosphate dehydratase* (DEP1), *tyrosine aminotransferase*, *5’-methylthioadenosine nucleosidase* (MTN), *adenylyl-sulfate reductase* (APR), *5-methyltetrahydropteroyltriglutamate-homocysteine methyltransferase* (metE), *alanine-glyoxylate transaminase* (AGXT2), *methylthioribose-1-phosphate isomerase* (mtnA), and *aspartate aminotransferase* (ASP5) were identified as mainly related to sulfur, cysteine, and methionine metabolism at the transcriptome level. Additionally, cysteine synthase and malate dehydrogenase (MDH1) showed the same up-regulated pattern at both the transcriptomic and proteomic levels (**Figure 5**). The majority of the DEGs were up-regulated at the transcriptome level, except *adenylyl-sulfate reductase* (APR), *homocysteine S-methyltransferase* (mmuM), *DNA (cytosine-5)-methyltransferase1* (DNMT1), and *aminocyclopropanecarboxylate oxidase* (E1.14.17.4) (**Figure 5**).

## Discussion

Emerging evidence suggests that the versatile signal molecule H_2_S can ameliorate some of the adverse effects induced by salt stress. We have previously reported that exogenous application of H_2_S alleviates the NaCl-induced toxicity in the leaves and roots of cucumber through analysis of morphology, photosynthesis, stomatal responses, ROS accumulation, and H_2_S homeostasis at the physiological and biochemical levels (Jiang et al., 2019). The purpose of this study was to explore the potential regulatory mechanism underlying H_2_S involvement in response to salt stress in cucumber. Our analyses revealed significant changes in transcriptome and proteome levels and identified numerous DEGs and DEPs as well as the metabolic and signaling pathways that might be related to H_2_S regulation of salt-stress tolerance.

### Primary Metabolism and Energy Metabolism

Carbohydrate and energy metabolism are necessary for both plant growth and stress response. In this work, salt stress caused significant changes at the transcriptomic level in carbon metabolism (62 DEGs), biosynthesis of amino acids (59 DEGs), carbon fixation in photosynthesis (25 DEGs), nitrogen metabolism (16 DEGs), and fatty acid degradation (15 DEGs) (S vs. C, **Figure 2**). However, addition of H_2_S to the salt treatment resulted in the disappearance of these pathway compared to salt stress alone (H_2_S vs. S, **Figure 3**). The balance between protein synthesis and degradation also plays an essential role in the regulation of cellular processes in response to environmental cues Reinbothe et al., 2010; Rothman, 2010). Salt stress also caused significant changes in the levels of protein involved in photosynthesis (7 DEPs), carbohydrate and energy metabolism (7 DEPs), and protein folding, assembly, and degradation (5 DEPs) (S vs. C, **Figure S2B**). Addition of H_2_S to the salt treatment caused a decline in the number of DEPs compared to the number seen under salt stress alone (H_2_S vs. S, **Figure S2B**). This data is consistent with a previous report in sesame showing that salinity stress changed the abundance of many proteins involved in the biosynthesis and degradation of amino acids such as alanine, aspartate, valine, leucine, isoleucine, and glutamate (Zhang et al., 2019). These results indicated that salt stress caused significant changes in primary and energy metabolism, which might lead to plant growth inhibition. However, H_2_S could regulate these pathways to recover to levels similar to the control sample. Plants need energy to regulate a variety of processes, including ROS scavenging and protective substance synthesis, to prevent salt stress-induced damage, and adequate energy is crucial for cucumber roots to resist salt stress (Du et al., 2018).

Under salt stress, photosynthesis is impaired due to stomatal closure and limited carbon dioxide intake. Plants with more active resistance to stress always have more energy demands (Zhu, 2001; Kosova et al., 2011). In this study, a large number of DEGs or DEPs related to photosynthesis, such as *ribulose-phosphate 3-epimerase*, chloroplastic (Csa_1G038360; XP_004137418.1), *ribulose bisphosphate carboxylase small chain* (Csa_5G609710; O65194), *photosystem I reaction center subunit N* (Csa_6G483300), *chlorophyll a-b binding protein 6A* (Csa_5G490820), *photosynthetic NDH subunit of lumenal location 3*(Csa_3G414060), *carbonic anhydrase 2 isoform X2* (XP_004141091.2), *chloroplast stem-loop binding protein of 41 kDa a* (XP_004140080.1), and *rubisco accumulation factor 1* (XP_004142574.1), were detected at the transcriptomic or proteomic level during H_2_S regulation of salt-stress tolerance in cucumber (**Tables 1** and **S2**). In agreement with this observation, Jiang et al. (2019) found significant declines in the photosynthetic parameters P_n_, T_r_, and F_v_/F_m_ and in the chlorophyll content (C_a_, C_b_, C_a+b_, and C_x+c_) under salt stress, and that H_2_S alleviated these declines (Jiang et al., 2019).

Carbohydrate and nitrogen metabolism are complex phenomena driven by the coordinate expression of numerous genes (Zhao et al., 2019). Our transcriptome data showed that numerous DEGs involved in carbohydrate metabolism, such as *malate synthase*, *glyoxysomal* (Csa_1G050360), *NADP-dependent glyceraldehyde-3-phosphate dehydrogenase* (Csa_2G009470), *citrate synthase* (Csa_2G012660), *formate dehydrogenase* (Csa_3G836500), *acyl-CoA dehydrogenase family member 10* (Csa_3G164520), and *fructose-bisphosphate aldolase cytoplasmic isozyme* (Csa_3G750920), and some DEGs involved in nitrogen metabolism, such as *nitrate reductase [NADH]-like* (Csa_4G377160) and *cyanate hydratase* (Csa_3G134790), responded to H_2_S regulation of salt-stress tolerance in cucumber (**Table S2**). In the proteomic data, metabolism-related proteins likely corresponding to these DEGs, namely, 6-phosphogluconolactonase 4 (XP_004145948.1), ribulose bisphosphate carboxylase (XP_004138462.1), ketol-acid reductoisomerase (XP_004136850.1), acetylornithine deacetylase (XP_004148484.1), malate dehydrogenase (XP_004147146.1), pyruvate dehydrogenase E1 component subunit beta-1 (XP_011656794.1), glyceraldehyde-3-phosphate dehydrogenase (XP_004152516.1), and sedoheptulose-1,7-bisphosphatase (NP_001267658.1), also responded to H_2_S regulation of salt-stress tolerance in cucumber (**Table 1**). In sesame, proteins related to energy metabolism and amino acid biosynthesis and metabolism were also salt-responsive (Zhang et al., 2019).

### Proton and Ion Transport Under Salt Stress

In this study, *V-type proton ATPase subunit a2* (Csa_2G364570) and *cation/H^+^ antiporter15/18* (Csa_3G563310; Csa_4G050260) were both detected in the transcriptomic data, indicating that H_2_S might regulate proton and ion transport under salt-stress (**Table S2**). Removing cytosolic Na^+^ from plant cell generally through the activities of Na^+^/H^+^ antiporters is critically important for plants to survive under salt stress. Additionally, previous research has shown that the shaker AKT1-like channels are likely involved in both high- and low-affinity K^+^ uptake and correlate with salt tolerance in plants (Duan et al., 2015). Our transcriptome data showed that *potassium channel AKT1* (Csa_1G303700) was involved in H_2_S regulation of salt-stress tolerance in cucumber (**Table S2**).

### Metabolic Pathways of Sulfur-Containing Compounds

Sulfur-containing compounds, including cysteine, methionine, glutathione, thioredoxin, and H_2_S, are crucial for the survival of plants under biotic and abiotic stress. Cysteine exists at the cross pathway of primary metabolism, protein synthesis, and the formation of low M_r_ sulfur-containing defense compounds (Rausch et al., 2005). Our data revealed that H_2_S treatment resulted in significant changes in cysteine and methionine metabolism (21 DEGs) and glutathione metabolism (20 DEGs) at the transcriptomic level (H_2_S vs. C, **Figure 3**). Sulfur-containing compounds impact plant defense, and their formation saturates at a higher S-supply than does plant growth (Bloem et al., 2005). Our transcriptome analysis showed that numerous DEGs related to metabolism of sulfur-containing compounds, including *probable sulfate transporter 3.5* (Csa_1G378520), *thioredoxin H-type/M-typ* (Csa_3G865340; Csa_6G343710), *thioredoxin-like 3-2* (Csa_5G622670), *cysteine-rich repeat secretory protein 3* (Csa_2G171840), and *methionine S-methyltransferase* (Csa_3G113310), responded to the addition of H_2_S during salt stress in cucumber (**Table S2**). In addition, H_2_S homeostasis in plant cells is closely related to cysteine metabolism. Similarly, application of H_2_S also caused significant changes at proteome level in sulfur, cysteine and methionine metabolic pathways (3 DEPs) and glutathione metabolism (1 DEP) (**Figure S3**). Cysteine synthase 1 (XP_004138495.1), glutathione S-transferase U25-like (XP_004147762.1), and protein disulfide-isomerase (XP_004149798.1) were observed through proteomic analysis (**Table 1**). H_2_S homeostasis is closely regulated by L-/D-cysteine desulfhydrase and L/D-cystiene desulphhydrase can catalyze the degradation of L-/D-cysteine to produce H_2_S, amine and pyruvate. It is surprising that L/D-cystiene desulphhydrase is not detected through transcriptome and proteome sequencing. However, Jiang et al. (2019) demonstrated that the activities of D/L-cysteine desulfhydrase significantly increased under excess NaCl and the endogenous H_2_S level also increased (Jiang et al., 2019). The metabolism related to sulfur-containing compounds, especially those that control the endogenous H_2_S level by regulating H_2_S synthetic and decomposition enzymes, plays a very important role in cucumber during H_2_S regulation of salt-stress tolerance.

### Plant Hormone Signal Transduction-Related Pathways

Perception and transmission of signals would be the first steps of a response within cucumber leaves to H_2_S regulation of salt-stress tolerance, and these interactions form a network of regulation leading to a variety of physiological responses (Sang et al., 2016). Here, several signal transduction-related pathways, such as plant hormone signal transduction (32 DEGs) and cAMP signaling pathway (6 DEGs), significantly responded at the transcriptomic level with application of H_2_S (H_2_S vs. S, **Figure 3**). Although no plant hormone signal transduction-related protein was found among the differentially accumulated proteins, numerous plant hormone signal transduction-related genes, such as *auxin-responsive protein IAA13* (Csa_3G134550), *auxin-binding protein ABP19a* (Csa_7G450510), *ethylene-responsive transcription factor ERF017* (Csa_1G597730), *salicylic acid-binding protein 2* (Csa_6G490080), *abscisic acid receptor PYR1-like* (Csa_3G902310), and *gibberellin-regulated protein* (Csa_3G826660), were detected in the DEG data (**Table S2**). Ethylene-, abscisic acid-, and salicylic acid-induced signal transduction pathways are frequently reported to be closely related to plant resistance to various stresses. Additionally, calcium signals are involved in plant responses to various stimuli, including abiotic and biotic stresses, and regulate a wide range of physiological processes (Harper, 2001). Our data showed that *calcium sensing receptor* (Csa_1G041560), *calcium-dependent protein kinase 32* (Csa_4G172540), *putative* *calcium-binding protein CML19* (Csa_1G002110), *calcium-binding protein PBP1-like* (Csa_1G071800), *calcium uniporter protein 3*, *mitochondrial-like* (Csa_6G487650), *calcium permeable stress-gated cation channel 1* (Csa_2G416110), *calcium-transporting ATPase4* (Csa_5G310810), and *cation/calcium exchanger 2-like* (Csa_3G822350) were detected in the DEG data during H_2_S regulation of salt-stress tolerance in cucumber (**Table S2**). H_2_S has been reported to alleviate oxidative damage under excess nitrate stress through mitogen-activated protein kinase signaling in cucumber (Qi et al., 2019). As expected, *mitogen-activated protein kinase 3-like* (Csa_1G479630) and *cyclic nucleotide-gated ion channel 1-like* (Csa_3G607120) as well as a few *transcription factor IBH1/BIM1/MYB86-like/RAX3* genes (Csa_1G294080; Csa_1G015080; Csa_1G046820; Csa_2G352410) were also detected in the transcriptomic data during H_2_S regulation of salt-stress tolerance in cucumber (**Table S2**). Moreover, the proteome data showed that inositol phosphate metabolism (Inositol-3-phosphate synthase-like; XP_004149141.2), an important signal transduction pathway, was involved in H_2_S regulation of salt-stress tolerance in cucumber.

### Defense Related Pathways

Plants suffering from NaCl toxicity often exhibit symptoms associated with oxidative stress and membrane lipid peroxidation, which can result in accumulation of reactive oxygen species and malondialdehyde (Hossain et al., 2005). Thus, under various biotic and abiotic stresses, stress- and defense-related proteins have been frequently reported (Liu et al., 2018). Under salt stress, the genes *L-ascorbate peroxidase* (Csa_6G507100), *peroxidase P7* (Csa_1G586250), *superoxide dismutase* *[Fe]/[Mn]/[Cu-Zn]* (Csa_1G571850; Csa_1G025980; Csa_2G013250), and *catalase isozyme 1* (Csa_4G658590) were significantly up-regulated and the *L-ascorbate oxidase* were significantly down-regulated with the transcriptome sequencing. However, the genes *L-ascorbate peroxidase* (Csa_6G507100), *peroxidase P7* (Csa_1G586250), and *superoxide dismutase [Cu-Zn]* (Csa_2G013250) were significantly down-regulated with the transcriptome sequencing, when the exogenous H_2_S was applied the cucumber under salt stress (**Table S2**). Consistent with the transcriptome analysis, peroxidase (AAA33129.1) and glutathione S-transferase U25-like (XP_004147762.1) were also detected in the proteomic data (**Table 1**). Peroxidase, which can also catalyze the reduction of H_2_O_2_, was induced by salt stress (Yan et al., 2005). Glutathione S-transferases (XP_004147762.1) represent a major sample of detoxification enzymes that play important roles in protecting plants from impairments caused by abiotic stresses (Liu et al., 2019). Moreover, *heat shock 70 kDa protein* (Csa_4G617390) was detected in the transcriptomic data during H_2_S regulation of salt-stress tolerance in cucumber (**Table S2**). Heat shock proteins are also associated with the stress response and play an important role as chaperones, including in protein folding and degradation under stress conditions (Carmo et al., 2019).

## Conclusions

This study represents the first attempt to survey the complexity underlying H_2_S regulation of salt-stress tolerance in cucumber at the transcriptome and proteome levels. Interpretation of the transcriptomic and proteomic data uncovered a number of candidate genes and proteins that may be involved in H_2_S regulation of salt-stress tolerance mechanism of cucumber. These DEGs or DEPs were involved in both primary and energy metabolic pathways, such as photosynthesis, carbon metabolism, biosynthesis of amino acids, nitrogen metabolism, and fatty acid degradation, in sulfur-containing metabolic pathways and in cell defense and signal transduction pathways. Simultaneously, any correlation detected between changes in abundance in both transcripts and proteins may provide information regarding post-transcriptional control or time-dependent delays from transcript to protein during the H_2_S regulation of salt-stress tolerance in cucumber. Although much more work is needed to elucidate the specific functions of these identified genes and proteins, our findings will aid further research aiming to identify key genes and proteins active during H_2_S regulation of salt-stress tolerance.

## Data Availability Statement

The datasets generated for this study can be found in the NCBI SRA accession SRP220380. Data are available *via* ProteomeXchange with identifier PXD020642 (Website: http://www.ebi.ac.uk/pride.)

## Author Contributions

Conceived and designed the experiments: JJ. Performed the experiments: XR. Analyzed the data: RH, NY, WS, and YD. Wrote the paper: JJ. Prepared the manuscript: JJ, CJ, and LL. All authors contributed to the article and approved the submitted version.

## Conflict of Interest

The authors declare that the research was conducted in the absence of any commercial or financial relationships that could be construed as a potential conflict of interest.

## Abbreviations

*AGXT2*, Alanine-glyoxylate transaminase; *ALT*, Alanine transaminase; *APR*, Adenylyl-sulfate reductase; *ASP5*, Aspartate aminotransferase; *ATP*, Adenosine Triphosphate; *cAMP*, Cyclic Adenosine monophosphate; *CHCA*, α-Cyano-4-hydroxycinnamic acid; Cx+c, Carotenoid; *2-DE*, Two-dimensional gel electrophoresis; *DEGs*, Differentially expressed genes; *DEP-1*, Methylthioribulose 1-phosphate dehydratase; *DEPs*, Differentially expressed proteins; *DNMT1*, DNA, (cytosine-5)-methyltransferase1; *DTT*, Dithiothreitol; *Fv/Fm*, Ratio of variable to maximum fluorescence; *FBP*, Fructose-1,6-bisphosphatase I; *GAPA*, Glyceraldehyde-3-phosphate dehydrogenase (NADP+); *GOT1*, Glutamic oxaloacetic transaminase; *H_2_S*, Hydrogen sulfide; *IAM*, Iodoacetamide; *IEF*, Isoelectric focusing; *IPG*, Immobilized pH gradient; *KEGG*, Kyoto Encyclopedia of Genes and Genomes; *MAEB*, Malate dehydrogenase (oxaloacetate-decarboxylating)(NADP+); *MALDI-TOF*, Matrix-assisted laser desorption/ionization time of flight; *MDH1*, Malate dehydrogenase; *MTN*, 5’-methylthioadenosine nucleosidase; *NADP*, Nicotinamide adenine dinucleotide phosphate; *NCBI*, National Center of Biotechnology Information; *NSCCs*, Nonselective cation channels; *Pn*, Net photosynthetic rate; *PCKA*, Phosphoenolpyruvate carboxykinase (ATP); *PCA*, Principal Coordinate Analysis; *PMF*, Peptide mass fingerprinting; *PPC*, Phosphoenolpyruvate carboxylase; *ROS*, Reactive oxygen species; *SDS-PAGE*, Sodium dodecyl sulfate polyacrylamide gel electrophoresis; *RNA-seq*, RNA sequencing; *SPSS*, Statistical product and service solutions; *TCA*, Trichloroacetic acid; *TFA*, Trifluoroacetic acid; *TOF-MS*, Time-off-flight mass spectrometer; *Tr*, Transpiration rate.
