# Supplementary figures and images for: H2S Regulation of Metabolism in Cucumber in Response to Salt-Stress Through Transcriptome and Proteome Analysis
Source: Front Plant Sci. 2020 Aug 19;11:1283. doi: 10.3389/fpls.2020.01283 (PMC7466724; doi:10.3389/fpls.2020.01283)

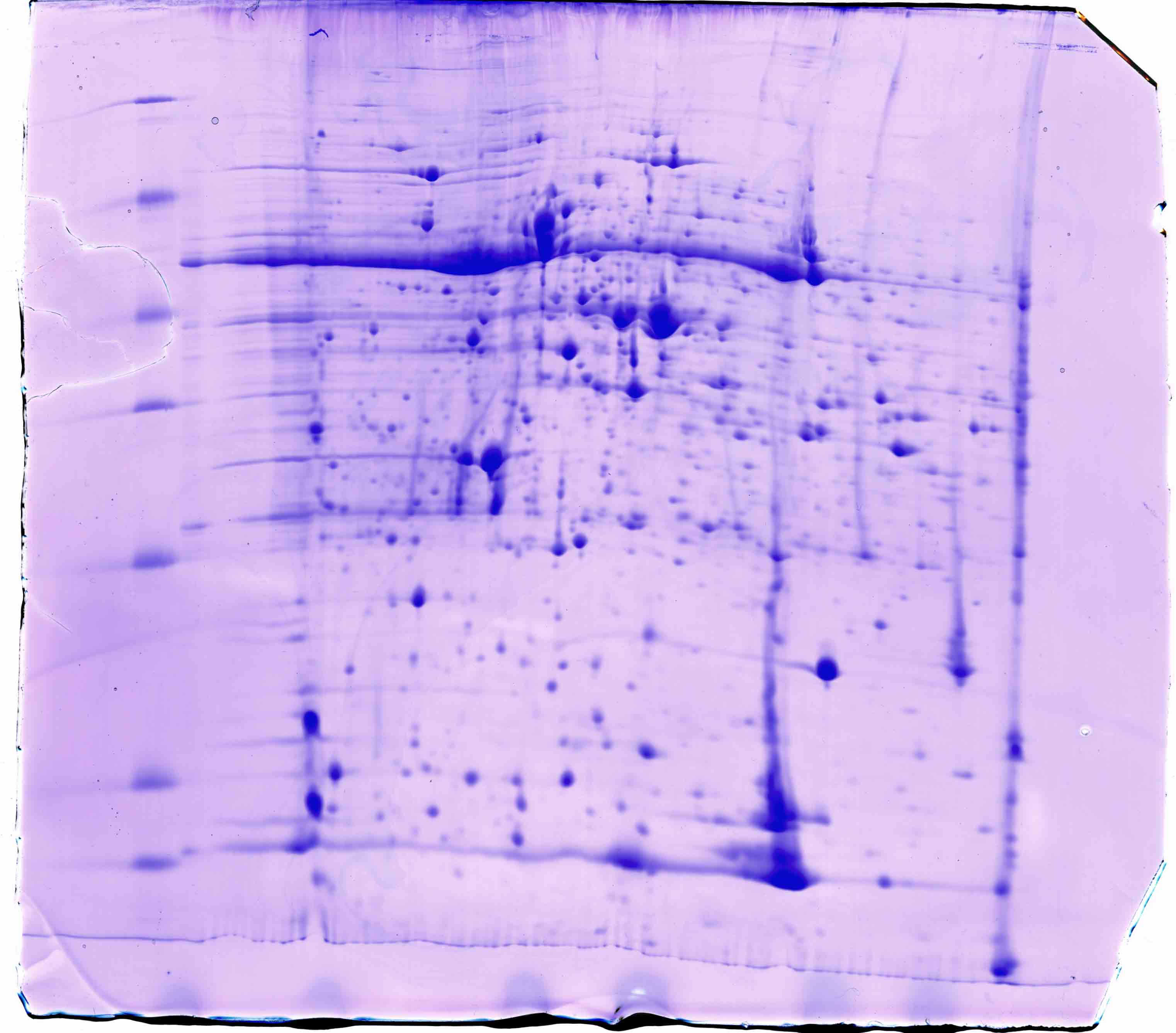

Supplement: Supplementary file 1 [file DataSheet_1.zip › Triplicate examples of 2D gels-1/Control group(C1-3ú⌐/CK-1.jpg]

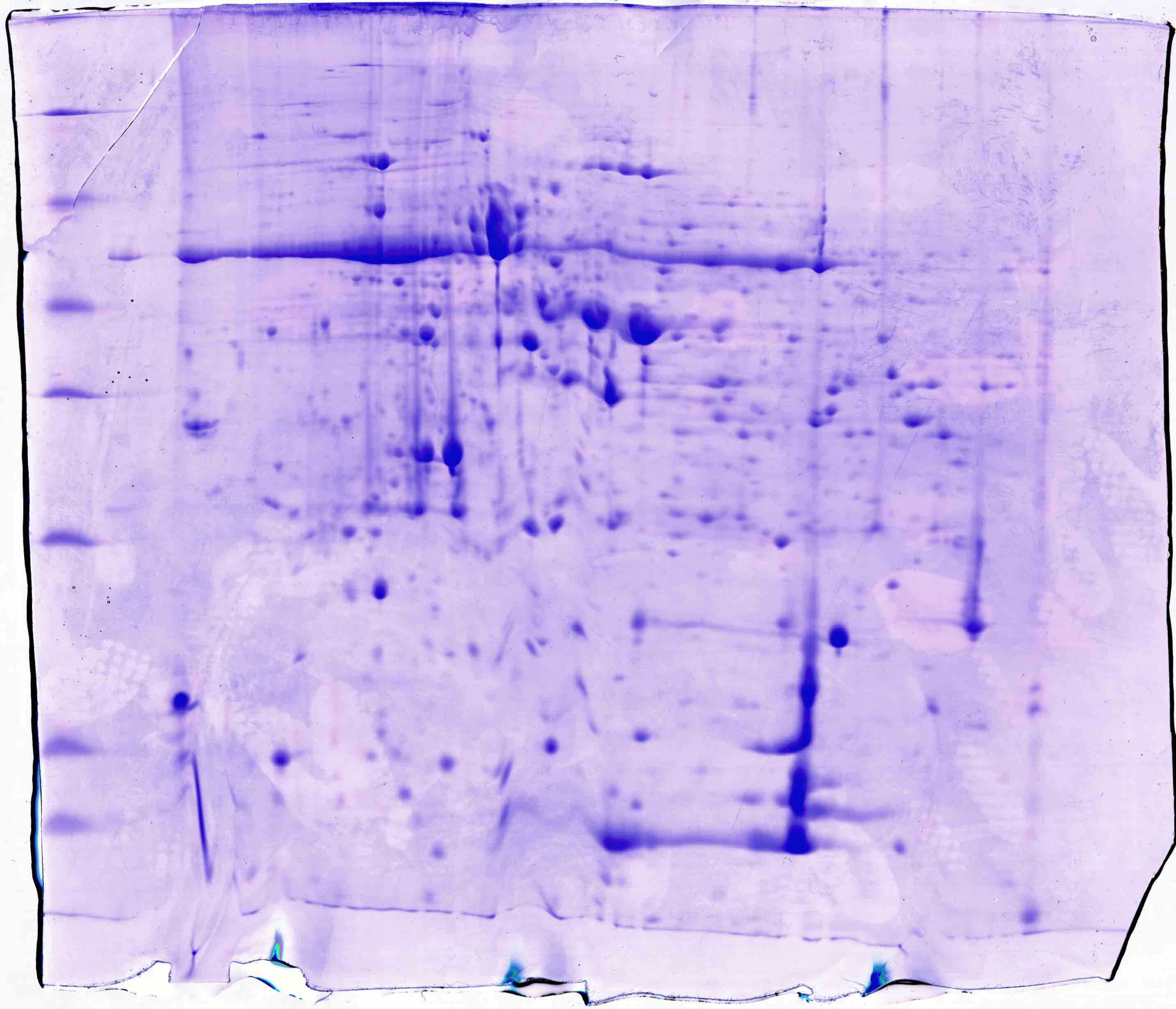

Supplement: Supplementary file 1 [file DataSheet_1.zip › Triplicate examples of 2D gels-1/Control group(C1-3ú⌐/CK-2.jpg]

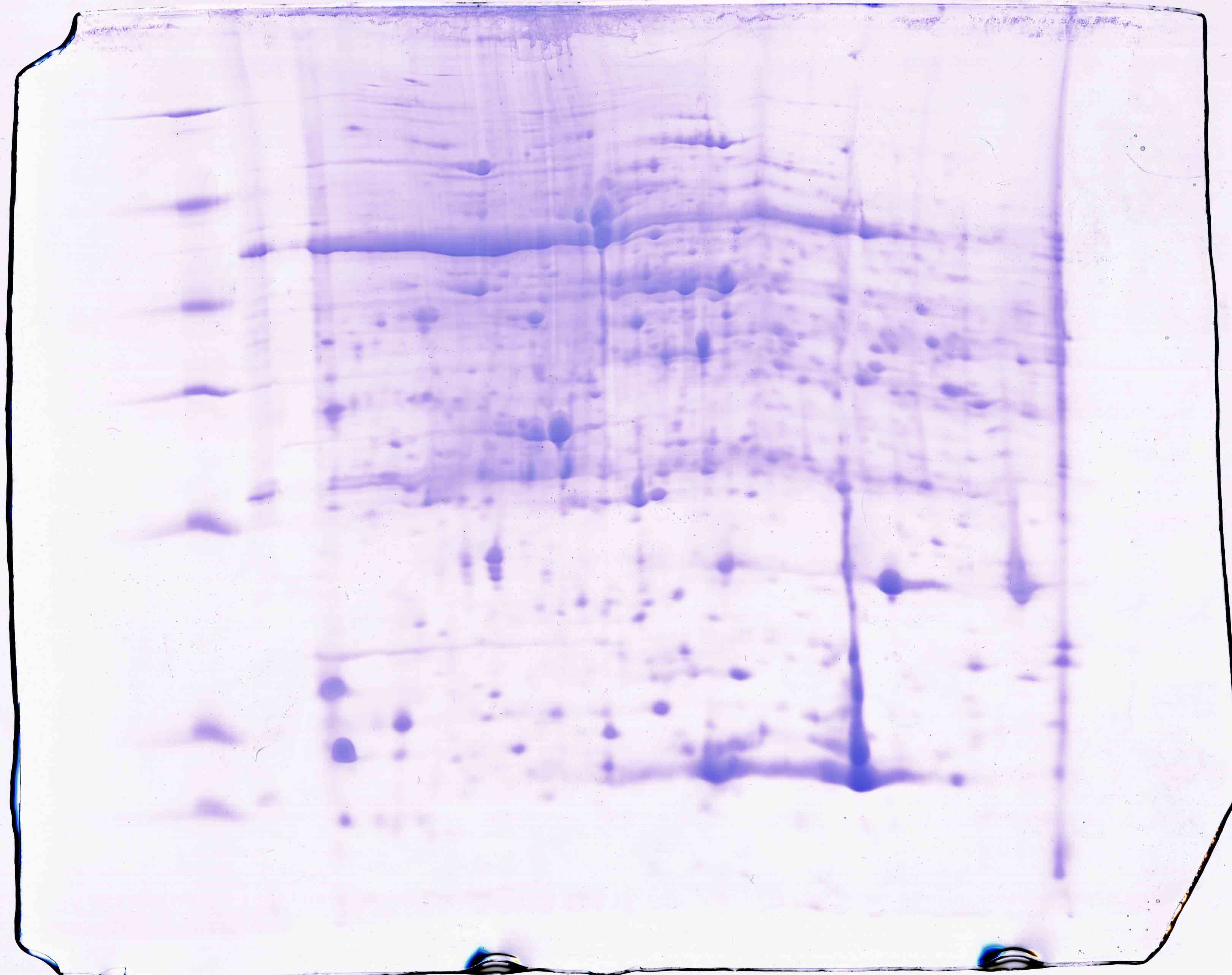

Supplement: Supplementary file 1 [file DataSheet_1.zip › Triplicate examples of 2D gels-1/Control group(C1-3ú⌐/CK-3.jpg]

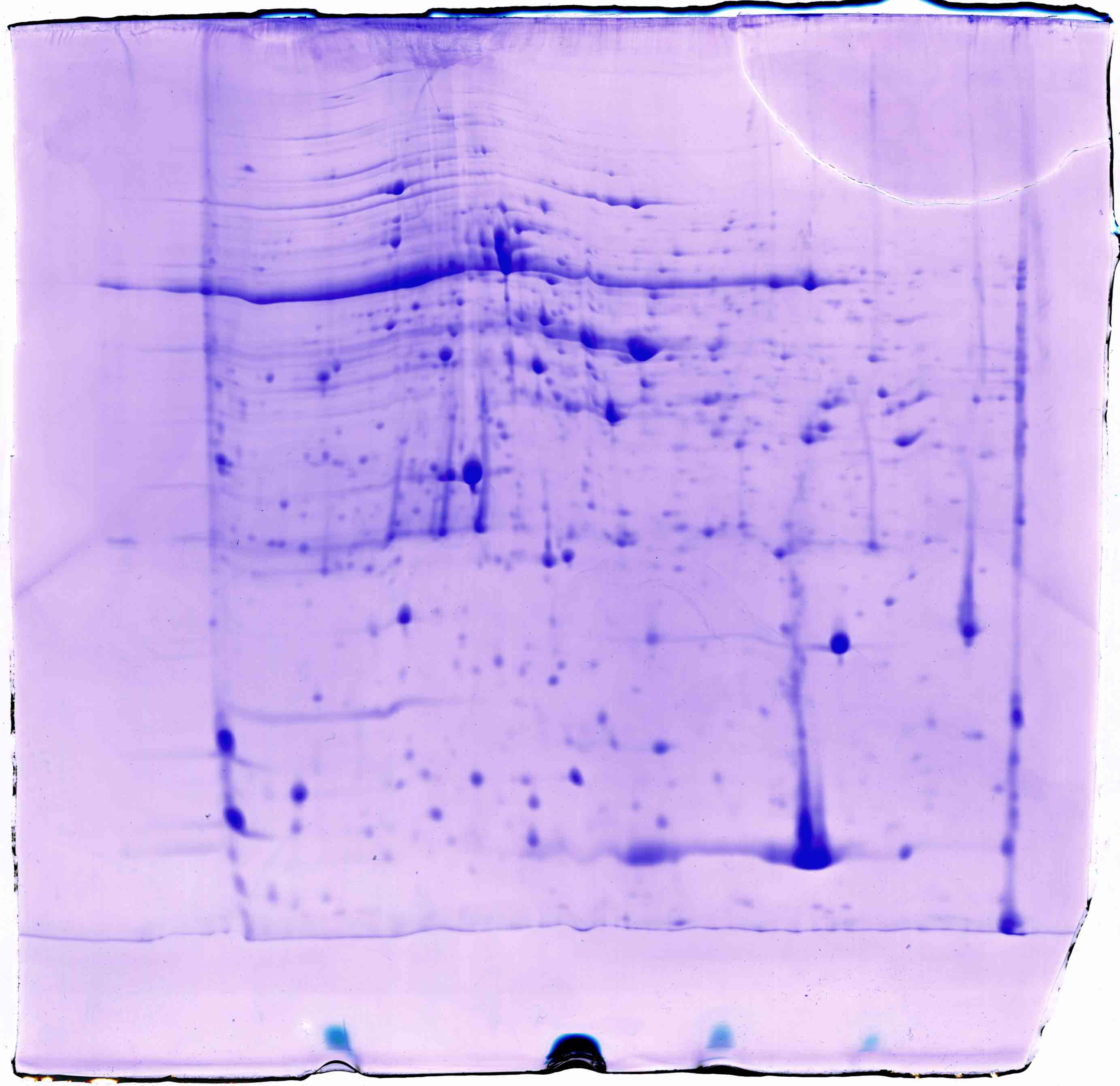

Supplement: Supplementary file 1 [file DataSheet_1.zip › Triplicate examples of 2D gels-1/H2S treatment group (H2S1-3)/H2S-1.jpg]

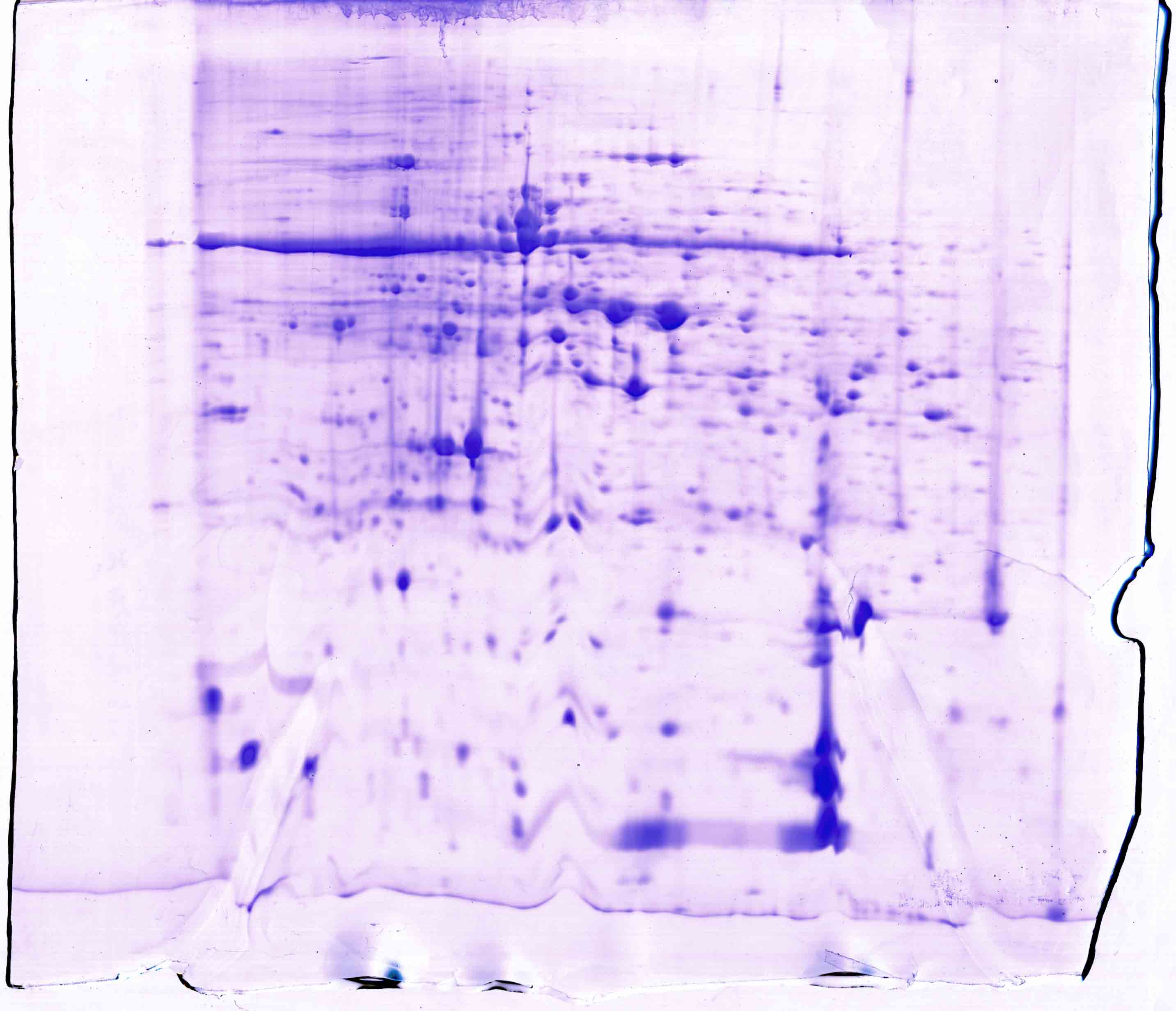

Supplement: Supplementary file 1 [file DataSheet_1.zip › Triplicate examples of 2D gels-1/H2S treatment group (H2S1-3)/H2S-2.jpg]

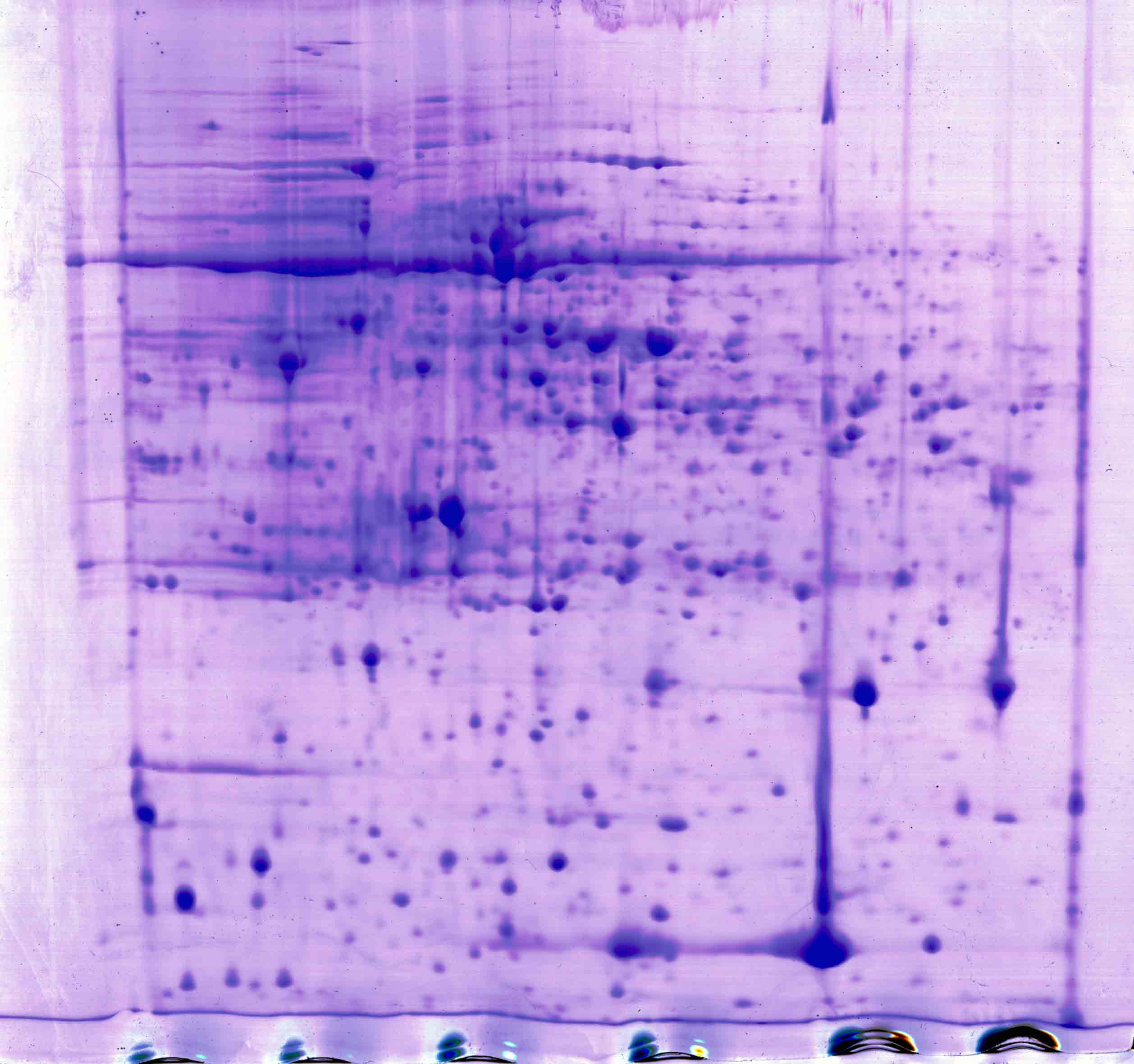

Supplement: Supplementary file 1 [file DataSheet_1.zip › Triplicate examples of 2D gels-1/H2S treatment group (H2S1-3)/H2S-3.jpg]

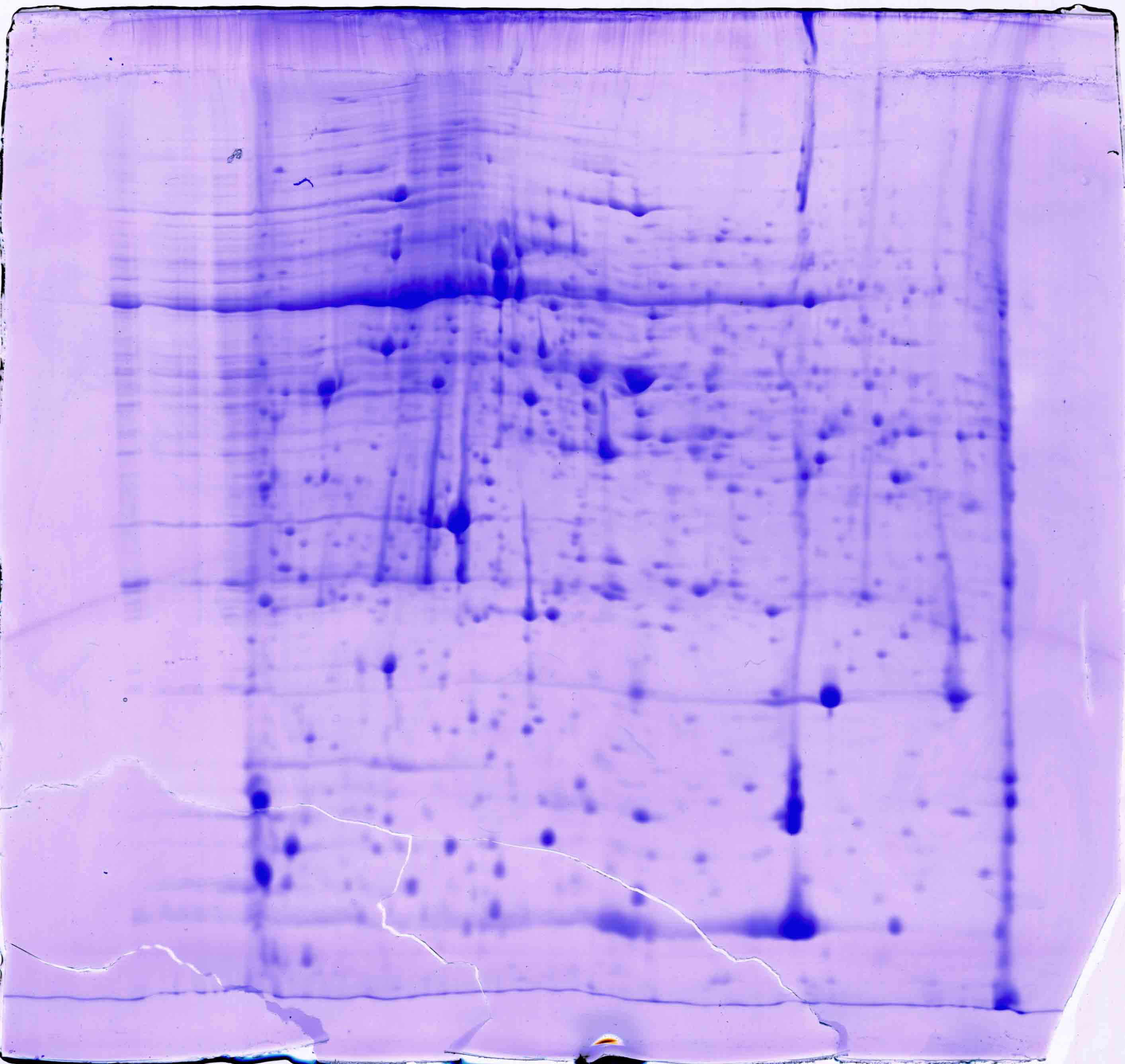

Supplement: Supplementary file 1 [file DataSheet_1.zip › Triplicate examples of 2D gels-1/Salt stress group(S1-3)/S-1.jpg]

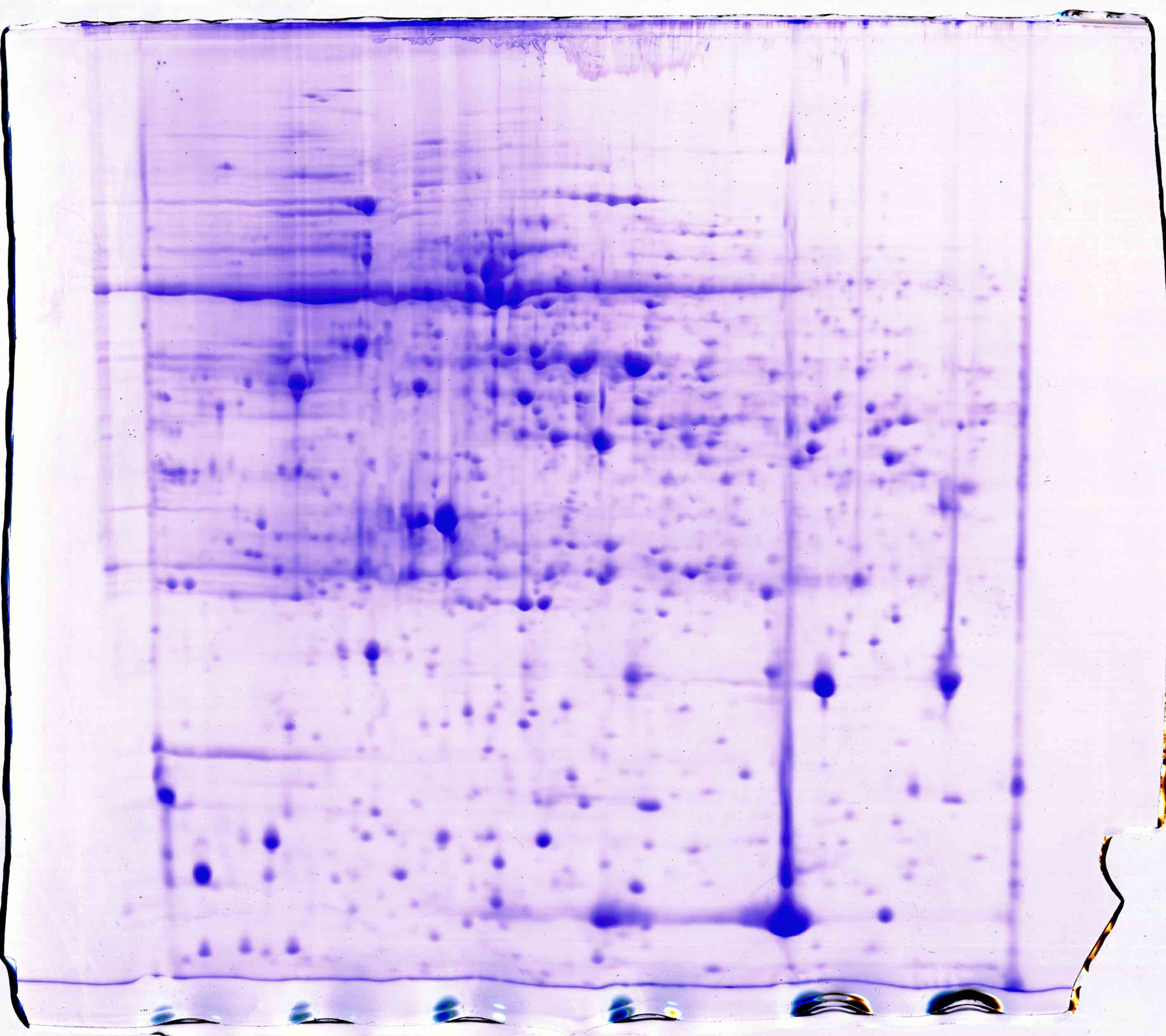

Supplement: Supplementary file 1 [file DataSheet_1.zip › Triplicate examples of 2D gels-1/Salt stress group(S1-3)/S-2.jpg]

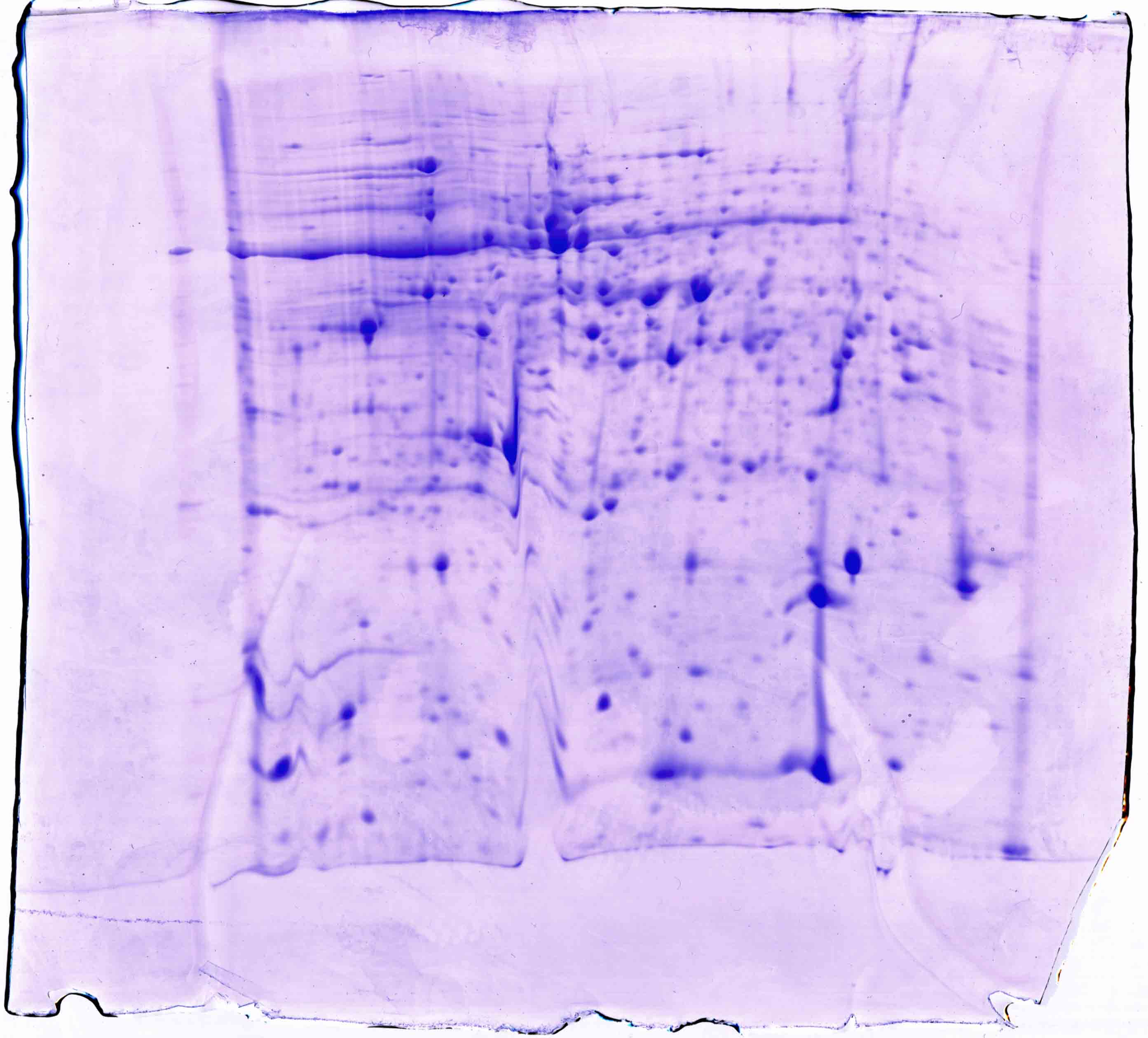

Supplement: Supplementary file 1 [file DataSheet_1.zip › Triplicate examples of 2D gels-1/Salt stress group(S1-3)/S-3.jpg]

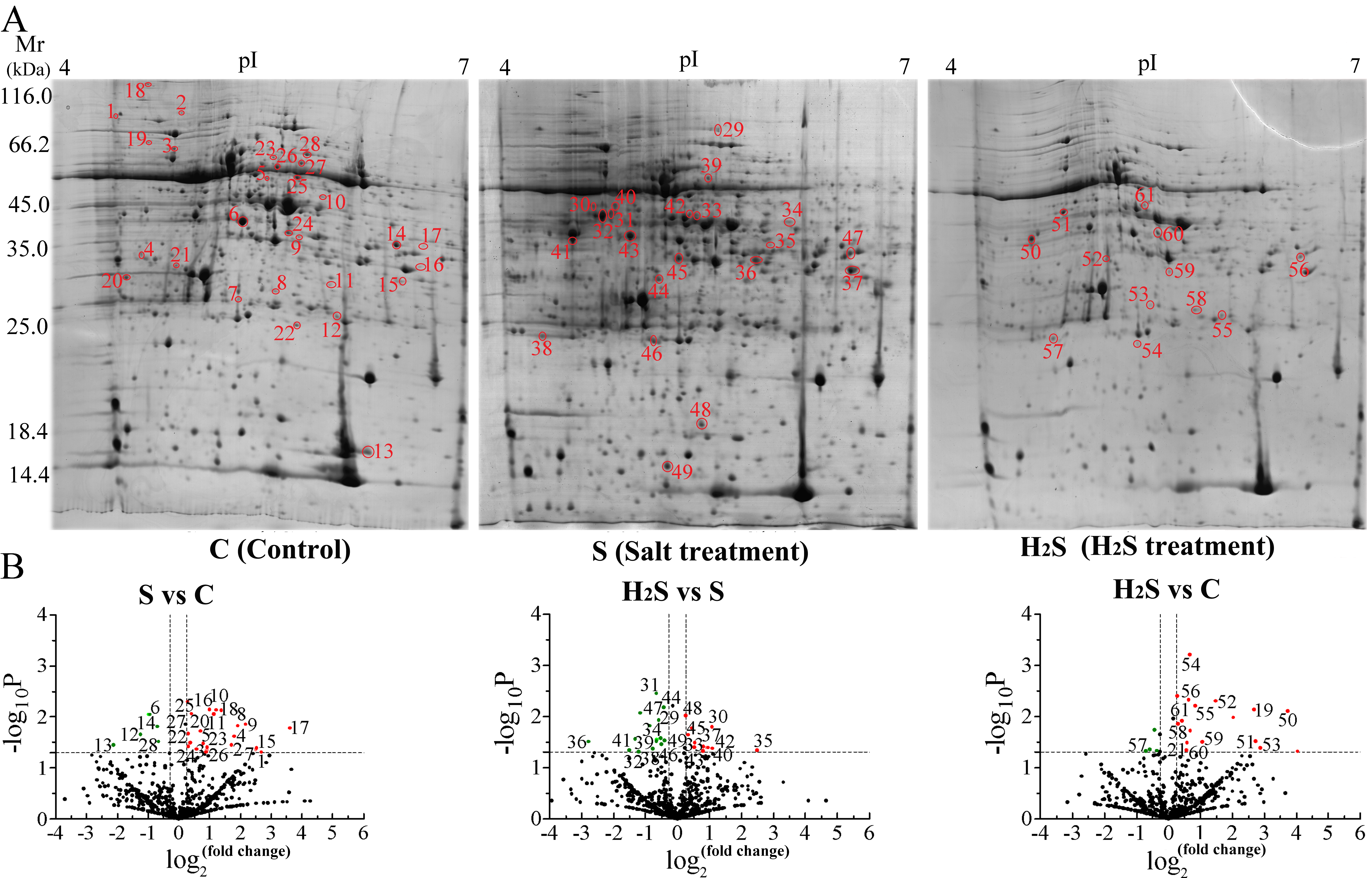

Supplement: Supplementary file 2 [file Image_1.tif]

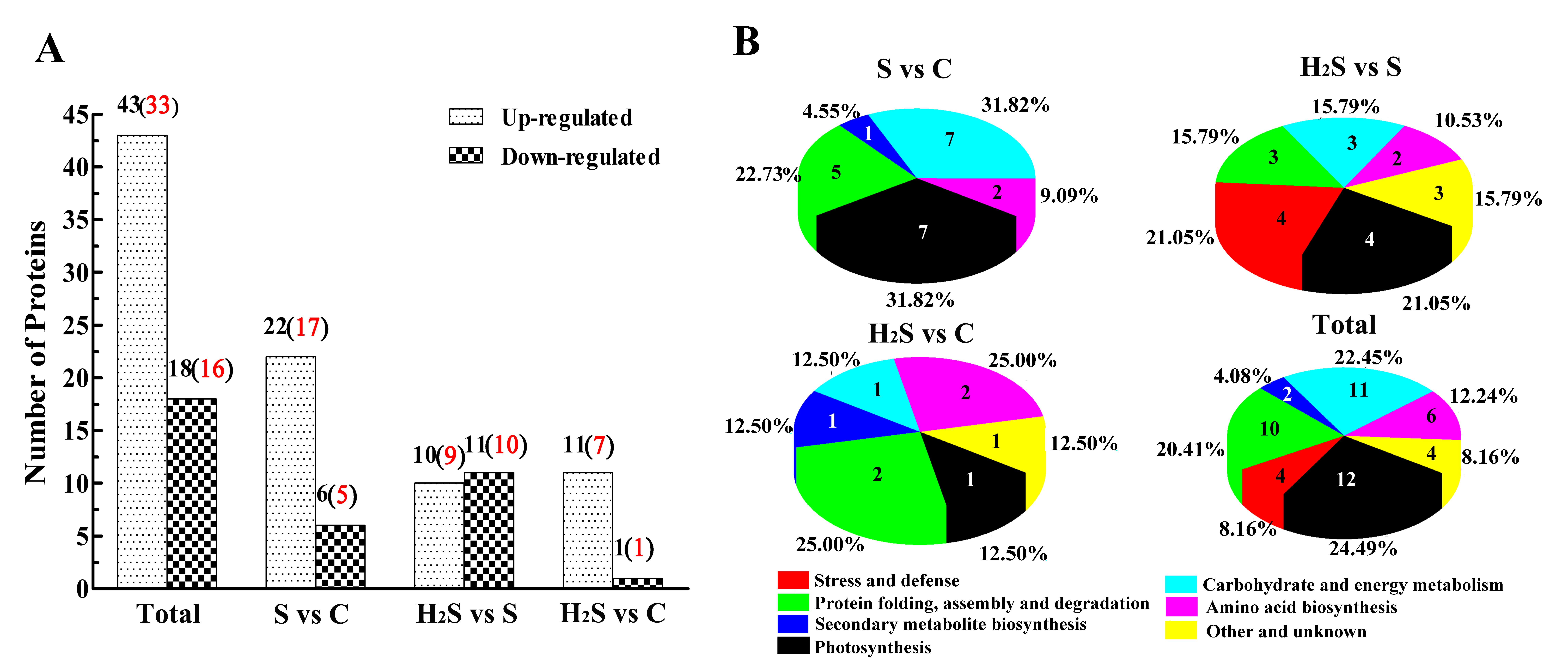

Supplement: Supplementary file 3 [file Image_2.tif]

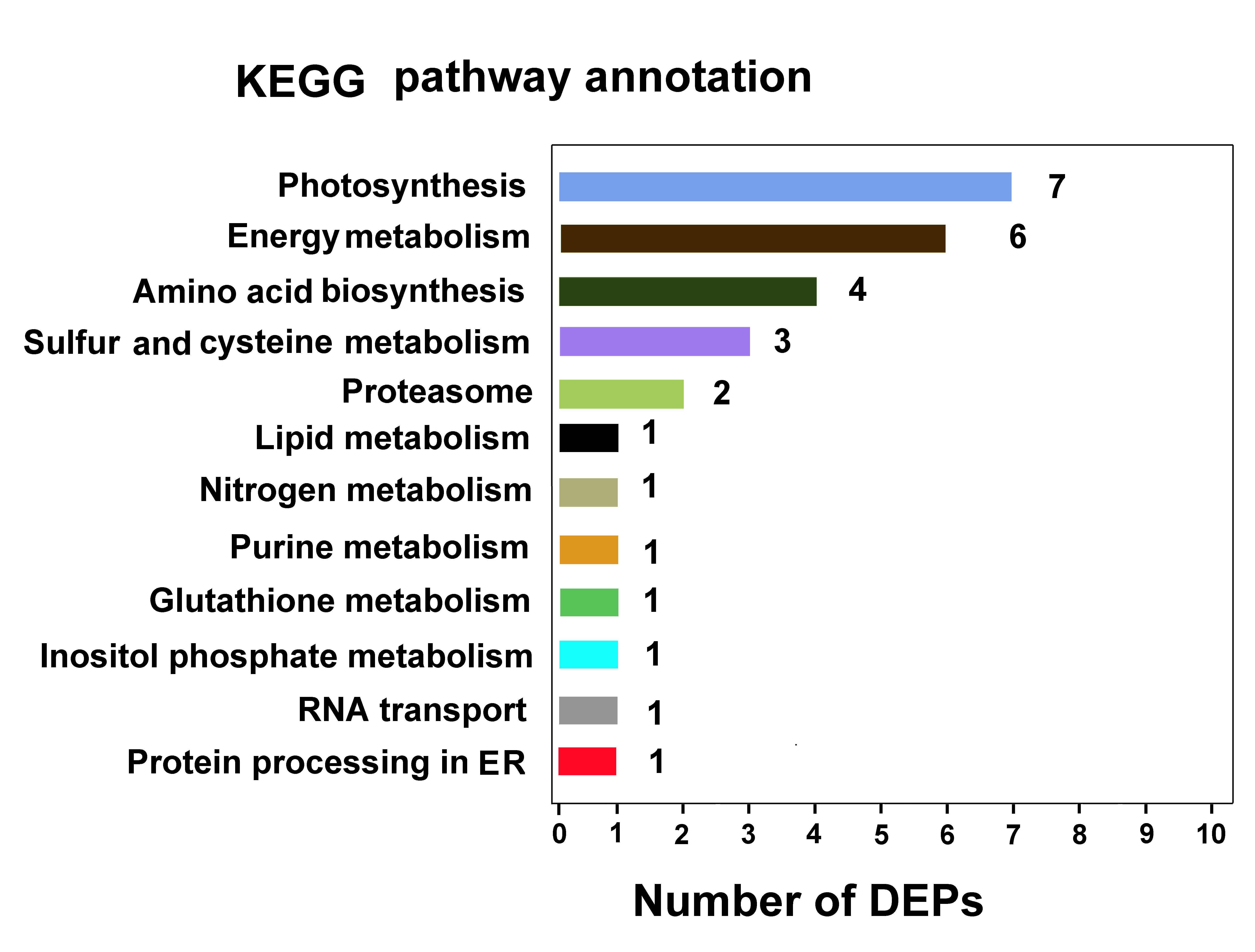

Supplement: Supplementary file 4 [file Image_3.tif]
