# Supplementary material for: H2S Regulation of Metabolism in Cucumber in Response to Salt-Stress Through Transcriptome and Proteome Analysis
Source: Front Plant Sci. 2020 Aug 19;11:1283. doi: 10.3389/fpls.2020.01283 (PMC7466724; doi:10.3389/fpls.2020.01283)
Supplement: Supplementary file 5 [file Table_1.doc]

**Table S1**

Summary for transcriptome sequencing data generated from nine cDNA libraries constructed from cucumber leaves following growth in Hoagland’s solution C, S and H_2_S.

| Sample | Raw Reads | Clean Reads | Q30 (%) | Total Mapped | Uniquely Mapped | Mapped to Gene |
| --- | --- | --- | --- | --- | --- | --- |
| C1 | 40607862 | 40095378  (98.73%) | 89.37% | 35542214  (88.64%) | 35129059  (87.61%) | 33723191  (96.00%) |
| C2 | 45736330 | 45015072  (98.42%) | 88.45% | 39462614  (87.67%) | 38993005  (86.62%) | 37383356  (95.87%) |
| C3 | 44482800 | 43335904  (97.42%) | 86.22% | 37160235  (85.75%) | 36742418  (84.79%) | 35459366  (96.51%) |
| S1 | 41562798 | 41246460  (99.23%) | 91.35% | 37444159  (90.78%) | 37079639  (89.90%) | 35696523  (96.27%) |
| S2 | 40664568 | 40244566  (98.96%) | 90.13% | 36043398  (89.56%) | 35630579  (88.54%) | 34183485  (95.94%) |
| S3 | 46421066 | 46095850  (99.29%) | 91.43% | 41810488  (90.70%) | 41304499  (89.60%) | 39659718  (96.02%) |
| H_2_S1 | 42706436 | 42271874  (98.98%) | 90.15% | 38092946  (90.11%) | 37738101  (89.27%) | 36344254  (96.31%) |
| H_2_S2 | 43135994 | 42571020  (98.69%) | 89.36% | 37751770  (88.68%) | 37272976  (87.55%) | 35873379  (96.25%) |
| H_2_S3 | 41932570 | 41326064  (98.55%) | 89.45% | 36880635  (89.24%) | 36533310  (88.40%) | 35182614  (96.30%) |

Q30: Percentage of sequences with sequencing error lower than 0.1 %.
