# Supplementary material for: H2S Regulation of Metabolism in Cucumber in Response to Salt-Stress Through Transcriptome and Proteome Analysis
Source: Front Plant Sci. 2020 Aug 19;11:1283. doi: 10.3389/fpls.2020.01283 (PMC7466724; doi:10.3389/fpls.2020.01283)
Supplement: Supplementary file 10 [file Table_6.doc]

**Table S6**

Statistical analysis of the pearson correlation between DEGs and DEPs

| Protein ID^a^ | Fold change^b^ | log2(fc) | Gene ID^c^ | Chromosome^d^ | Ratio^e^ | log2(R) | Direction^f^ | Description |
| --- | --- | --- | --- | --- | --- | --- | --- | --- |
| O65194 | 6.41 | 2.680 | Csa_5G609710 | 5 | 0.33 | -1.594 | + | ribulose bisphosphate carboxylase small chain,chloroplas- tic-like |
| XP_004149798.1 | 1.91 | 0.934 | Csa_6G383520 | 6 | 3.80 | 1.927 | + | protein disulfide-isomerase |
| Q9SAD7 | 3.63 | 1.860 | Csa_2G263980 | 2 | 1.49 | 0.571 | + | eukaryotic translation initiation factor 4B-like |
| XP_004139931.1 | 1.75 | 0.807 | Csa_6G133730 | 6 | 1.66 | 0.732 | + | 26S protease regulatory subunit 6B homolog |
| XP_004144412.1 | 0.52 | -0.943 | Csa_3G638540 | 3 | 0.51 | -0.958 | - | phosphoribulokinase, chloroplastic |
| XP_004145948.1 | 5.71 | 2.513 | Csa_5G139140 | 5 | 1.19 | 0.245 | + | probable 6-phosphogluconolactonase 4, chloroplastic |
| XP_004136700.2 | 3.78 | 1.918 | Csa_3G778270 | 3 | 6.62 | 2.727 | + | secoisolariciresinol dehydrogenase-like |
| XP_004154254.1 | 5.47 | 2.452 | Csa_2G234520 | 2 | 14.82 | 3.890 | - | probable carboxylesterase 13 |
| NP_001267500.1 | 2.33 | 1.220 | Csa_5G580630 | 5 | 1.07 | 0.096 | + | alpha-galactosidase-like |
| XP_004141091.2 | 2.21 | 1.144 | Csa_3G836520 | 3 | 0.40 | -1.319 | - | carbonic anhydrase 2 |
| XP_004137418.1 | 0.42 | -1.252 | Csa_1G038360 | 1 | 0.47 | -1.104 | + | ribulose-phosphate 3-epimerase, chloroplastic |
| ABN41481.1 | 0.23 | -2.120 | Csa_5G609710 | 5 | 0.33 | -1.594 | + | ribulose bisphosphate carboxylase small chain,chloroplas tic-like |
| XP_004140080.1 | 0.62 | -0.690 | Csa_6G408170 | 6 | 0.66 | -0.604 | + | chloroplast stem-loop binding protein of 41 kDa a,chloro- plastic |
| XP_004140080.1 | 12.34 | 3.625 | Csa_6G408170 | 6 | 0.66 | -0.604 | + | chloroplast stem-loop binding protein of 41 kDa a,chloro- plastic |
| XP_011652060.1 | 1.64 | 0.714 | Csa_1G050420 | 1 | 0.77 | -0.371 | - | elongation factor 1-delta |
| XP_004143844.1 | 1.48 | 0.566 | Csa_5G148580 | 5 | 2.04 | 1.029 | - | phosphoribosylformylglycinamidine cyclo-ligase,chloro- plastic/mitochondrial |
| XP_004141139.1 | 1.95 | 0.963 | Csa_3G843780 | 3 | 1.67 | 0.736 | + | T-complex protein 1 subunit theta |
| XP_004147146.1 | 1.51 | 0.595 | Csa_2G174150 | 2 | 1.48 | 0.562 | + | malate dehydrogenase, cytoplasmic |
| YP_004849346.1 | 1.34 | 0.422 | Csa_3G128910 | 3 | 1.67 | 0.742 | + | ATP synthase subunit delta', mitochondrial |
| XP_004143301.1 | 1.93 | 0.949 | Csa_6G450370 | 6 | 3.87 | 1.954 | + | enolase |
| XP_004149141.2 | 1.16 | 0.214 | Csa_4G031000 | 4 | 8.69 | 3.119 | - | inositol-3-phosphate synthase-like |
| XP_004136850.1 | 0.64 | -0.644 | Csa_7G051370 | 7 | 3.69 | 1.882 | - | ketol-acid reductoisomerase, chloroplastic |
| KGN63436.1 | 0.66 | -0.599 | Csa_1G000600 | 1 | 1.23 | 0.294 | + | - |
| XP_004142574.1 | 2.14 | 1.098 | Csa_1G690280 | 1 | 1.76 | 0.814 | - | rubisco accumulation factor 1, chloroplastic |
| XP_004148484.1 | 0.63 | -0.667 | Csa_3G902910 | 3 | 0.75 | -0.424 | + | acetylornithine deacetylase-like |
| XP_004148484.1 | 0.44 | -1.184 | Csa_3G902910 | 3 | 0.75 | -0.424 | + | acetylornithine deacetylase-like |
| XP_004147353.1 | 1.77 | 0.824 | Csa_2G301530 | 2 | 1.04 | 0.051 | - | actin-7 |
| XP_004142149.1 | 0.14 | -2.837 | Csa_4G285740 | 4 | 0.19 | -2.396 | - | peroxidase 2 |
| XP_011650288.1 | 1.96 | 0.971 | Csa_6G525450 | 6 | 0.76 | -0.400 | + | ferredoxin--NADP reductase, root isozyme, chloroplastic |
| XP_004136034.1 | 0.59 | -0.761 | Csa_7G396430 | 7 | 0.98 | -0.036 | - | uncharacterized protein OsI_027940 |
| XP_004138462.1 | 2.29 | 1.195 | Csa_6G008680 | 6 | 1.67 | 0.738 | - | ribulose bisphosphate carboxylase/oxygenase activase, chloroplastic |
| AAA33129.1 | 0.37 | -1.434 | Csa_4G285730 | 4 | 0.68 | -0.547 | - | peroxidase 2-like |
| XP_004146811.1 | 1.95 | 0.963 | Csa_3G848120 | 3 | 1.09 | 0.129 | - | eukaryotic initiation factor 4A-8 |
| NP_001267658.1 | 1.79 | 0.840 | Csa_5G198220 | 5 | 1.40 | 0.482 | + | sedoheptulose-1,7-bisphosphatase, chloroplastic-like |
| KGN54020.1 | 0.9 | -0.152 | Csa_4G268040 | 4 | 1.16 | 0.215 | + | uncharacterized LOC101217229 |
| XP_011656794.1 | 1.47 | 0.556 | Csa_6G091990 | 6 | 0.96 | -0.054 | - | pyruvate dehydrogenase E1 component subunit beta-1, mitochondrial |
| XP_004147762.1 | 0.71 | -0.494 | Csa_4G064630 | 4 | 1.19 | 0.246 | - | glutathione S-transferase U25-like |
| XP_004152516.1 | 0.75 | -0.415 | Csa_1G050250 | 1 | 1.12 | 0.163 | + | glyceraldehyde-3-phosphate dehydrogenase,cytosolic -like |
| XP_011654558.1 | 1.2 | 0.263 | Csa_5G128260 | 5 | 1.60 | 0.676 | - | peptidyl-prolyl cis-trans isomerase A1 |
| XP_004148484.1 | 6.64 | 2.731 | Csa_3G902910 | 3 | 1365.35 | 10.415 | + | acetylornithine deacetylase-like |
| XP_004148579.1 | 2.81 | 1.491 | Csa_3G664560 | 3 | 0.24 | -2.041 | + | - |
| XP_004141710.1 | 1.81 | 0.856 | Csa_7G448880 | 7 | 2.43 | 1.283 | + | proteasome subunit alpha type-6 |
| XP_004138495.1 | 1.56 | 0.642 | Csa_6G004600 | 6 | 1.19 | 0.251 | + | bifunctional L-3-cyanoalanine synthase/cysteine synthase 1, mitochondrial |
| XP_004137828.1 | 0.62 | -0.690 | Csa_3G740070 | 3 | 1.74 | 0.802 | - | 29 kDa ribonucleoprotein, chloroplastic |
| KGN54259.1 | 1.24 | 0.310 | Csa_4G296130 | 4 | 3.36 | 1.747 | - | uncharacterized protein At5g02240 |
| BAJ23911.1 | 1.5 | 0.585 | Csa_5G630800 | 5 | 3.34 | 1.740 | - | quinone oxidoreductase-like protein At1g23740,chloropl- astic-like |
| XP_004141688.1 | 1.11 | 0.151 | Csa_7G450710 | 7 | 1.10 | 0.140 | + | eukaryotic initiation factor 4A-11 |

^a^ Protein accession number from the NCBInr and SwissProt database.

^b^ Data in the column represents the mean of two independent replicates.

^c^ Protein accession number from the NCBInr database.

^d^ The chromosomal location of the differentially expressed genes.

^e^ Data in the column represents the FPKM mean of three independent replicates.

^f^ +/-: Direction of base sequence, sense strand and antisense strand, respectively.
